# Engineering Synthetic Electron Transfer Chains from Metallopeptide Membranes

**DOI:** 10.1021/acs.inorgchem.3c02861

**Published:** 2023-12-21

**Authors:** Anthony Sementilli, Rolando F. Rengifo, Wei Li, Andrew M. Stewart, Katie L. Stewart, Umar Twahir, Youngsun Kim, Jipeng Yue, Anil K. Mehta, Jason Shearer, Kurt Warncke, David G. Lynn

**Affiliations:** ^†^Departments of Chemistry, ^‡^Biology, and ^§^Physics, Emory University, Atlanta, Georgia 30322, United States; ∥ Department of Chemistry, Trinity University, San Antonio, Texas 78212, United States

## Abstract

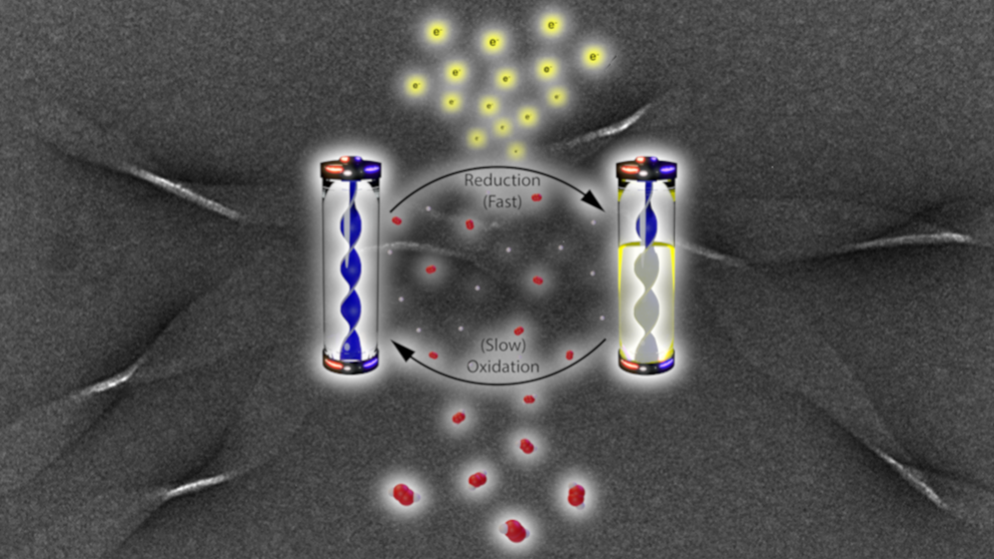

The energetic and geometric features enabling redox chemistry across the copper cupredoxin fold contain key components of electron transfer chains (ETC), which have been extended here by templating the cross-β bilayer assembly of a synthetic nonapeptide, HHQALVFFA-NH_2_ (K16A), with copper ions. Similar to ETC cupredoxin plastocyanin, these assemblies contain copper sites with blue-shifted (*λ*_*max*_ 573 nm) electronic transitions and strongly oxidizing reduction potentials. Electron spin echo envelope modulation and X-ray absorption spectroscopies define square planar Cu(II) sites containing a single His ligand. Restrained molecular dynamics of the cross-β peptide bilayer architecture support metal ion coordination stabilizing the leaflet interface and indicate that the relatively high reduction potential is not simply the result of distorted coordination geometry (entasis). Cyclic voltammetry (CV) supports a charge-hopping mechanism across multiple copper centers placed 10–12 Å apart within the assembled peptide leaflet interface. This metal-templated scaffold accordingly captures the electron shuttle and cupredoxin functionality in a peptide membrane-localized electron transport chain.

## Abstract 2

A simple peptide amphiphile with a metal-binding histidine dyad assembles in aqueous solutions of dilute Cu(II) to yield metalated peptide bilayers. These metallopeptide membranes organize their Cu(II) cofactors within sufficient proximity to facilitate electron transfer across micrometer regimes. Unlike MOFs, extensive quaternary structural contacts within the cross-beta scaffold affords a structural plasticity that maintains “membrane” integrity across multiple redox cycles via electrodes or substrate-level electron donors (i.e. ascorbate)−thus constituting a two-component biomolecular electron transfer console.

## Introduction

Electron transfer chains are fundamental to biochemistry. Most famously capitalized in mitochondrial oxidative phosphorylation^1^ and photosynthesis’s light-dependent reactions,^2^ life has established such conduits to power the proton gradients needed for ATP production.^3^ The source of this power (termed the “wireless current”)^4^ depends on successive, membrane-localized electron hops via redox-active shuttles^5,6^ from small molecules (e.g., ubiquinone) to entire metalloproteins (e.g., plastocyanin). This paradigm is biochemically ancient, efficient, and conserved^7−9^ but can inspire the design of new redox-active biomaterials.^10−14^ Past feats of crafting complex biomolecular systems range from polymerizing whole metalloproteins in vitro^15^ or in vivo^16^ to transforming bacterial outer membranes to respire electrodes.^17^ While elegant, such constructs are biosynthetically complex, and designing biomaterials that approximate natural electron transfer chains remains challenging. In this study, we present an array of copper ions and peptides that serve as a proof-of-concept for a self-assembled electron transfer console.

Minimally, an electron transfer chain can be abstracted to a membrane and a matrix of intrinsic redox shuttles. Our strategy simplifies these subcomponents into a single amphiphilic peptide: H-HHQALVFFA-NH_2_ (K16A).^18−21^ Containing the nucleating core of Aβ^22−24^ and a metal-binding His dyad, K16A autonomously forms dual-laminate structures reminiscent of phospholipid bilayers via 2-step nucleation.^25^ Unlike biological membranes, K16A membranes contain periodic metal cofactor pockets arrayed between the peptide leaflets.^18^ Extensive interrogations of these cross-β architectures, where peptide strands repeat perpendicularly to the aggregate’s growth axis,^26^ have revealed that buffer salts,^27^ the charge of the substituent peptides,^28^ and even mega-Dalton nucleic acids^25,29^ intercept and direct ensuing peptide membrane growth from the initial dynamic condensate.^20^ Here we show that Cu(II) ions template the K16A peptide into a scaffold amenable for multiple redox cycles across the bound metals and with the external environment, establishing a self-assembling system of biomolecular electron transfer at micron scales.

## Results and Discussion

### Metal Ion Templated Assembly

**Figure 1:**
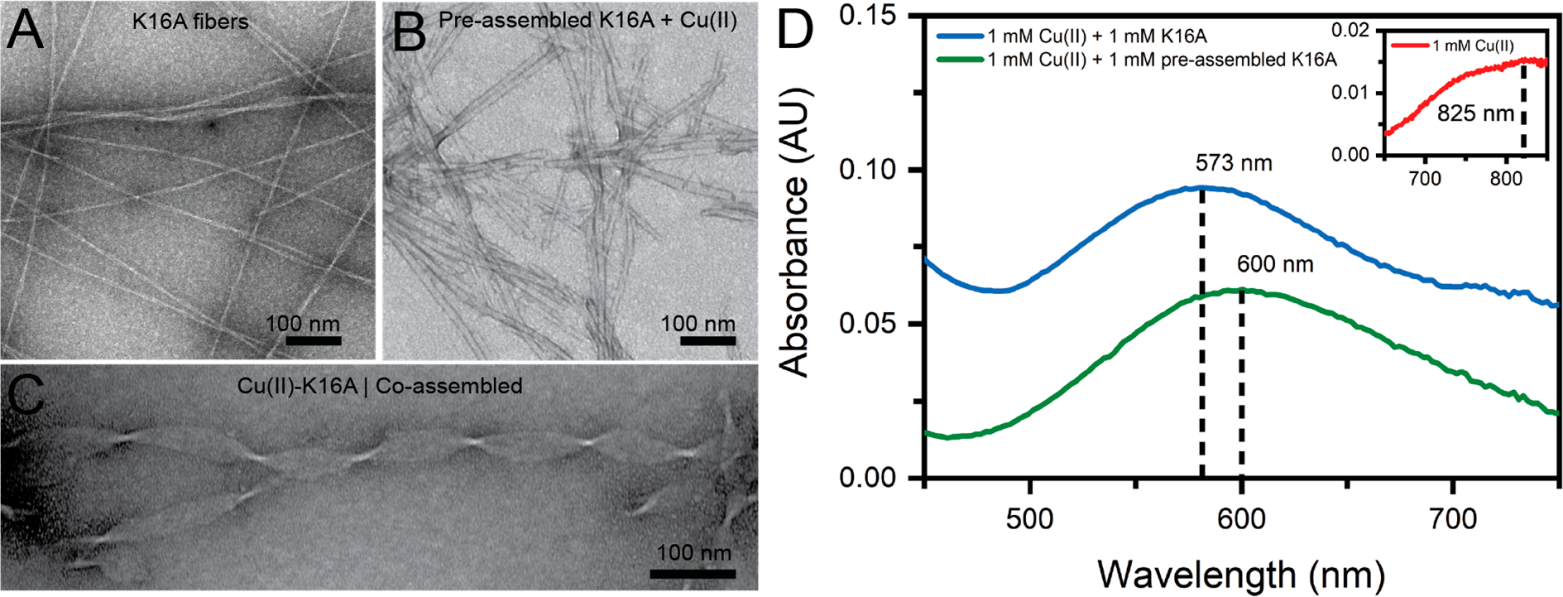
Electron micrographs of (A) preassembled K16A fibers, (B) preassembled K16A with added CuCl_2_, and (C) K16A coassembled with CuCl_2_ as “twisted ribbon membranes.” (D) Electronic spectra of coassembled Cu(II)-K16A “twisted ribbon membranes” (blue), preassembled K16A fibers + CuCl_2_ (green), and aqueous copper (red, inset). All samples were prepared in 25 mM MES, pH 5.6.

In the absence of metal ions, K16A assembles as fibrils via two-step nucleation (Figure 1A).^20^ Templated assembly of the initial metastable particle phase was first explored with a diverse range of transition metals, and the most effective metals were from the late first and second rows of the periodic table (Figure S1). CuCl_2_ was most notable, both in bundling the preassembled peptide fibrils into fibers (Figure 1B) and coassembling with equimolar K16A condensates to give morphologically distinct membrane-like structures resembling twisted ribbons (Figure 1C). Electron micrographs (TEM) of these “twisted ribbon membranes” enabled the definition of homogeneous 36.7 ± 0.3 nm widths maintaining a 304.4 ± 2.8 nm pitch (Figure S2). The presence of Cu(II) within these ribbon structures was confirmed by a significantly blue-shifted electronic transition *λ*_*max*_ at 573 nm relative to hexaaquacopper at *λ*_*max*_ 825 nm (Figure 1, insert), consistent with the stronger ligand-field interactions achieved by Cu(II) complexed with histidine side chains. When the preassembled K16A is titrated with Cu(II), a less blue-shifted transition appears, *λ*_*max*_ of 600 nm (Figure 1). These distinct membrane-like structures coassembled and preassembled with Cu(II) differ from assemblies of the previously studied full-length Aβ peptide where Cu(II) coordination appears independent of the degree of fibrillization.^30,31^ The differences in observed electronic transitions of all of these assemblies suggest distinct coordination modes. Basis sets derived from known square planar copper complexes have been used to predict ligand composition based on *λ*_*max*_(32) and indicate that (Im)_2_(ammino)_2_ and (Im)(ammino)(hydroxo)_2_ coordination give *λ*_*max*_ values consistent with the blue-shifted Cu(II)-K16A coassembled “membranes” (Table S1).

### Redox Cycling in Cu(II)-K16A

**Figure 2:**
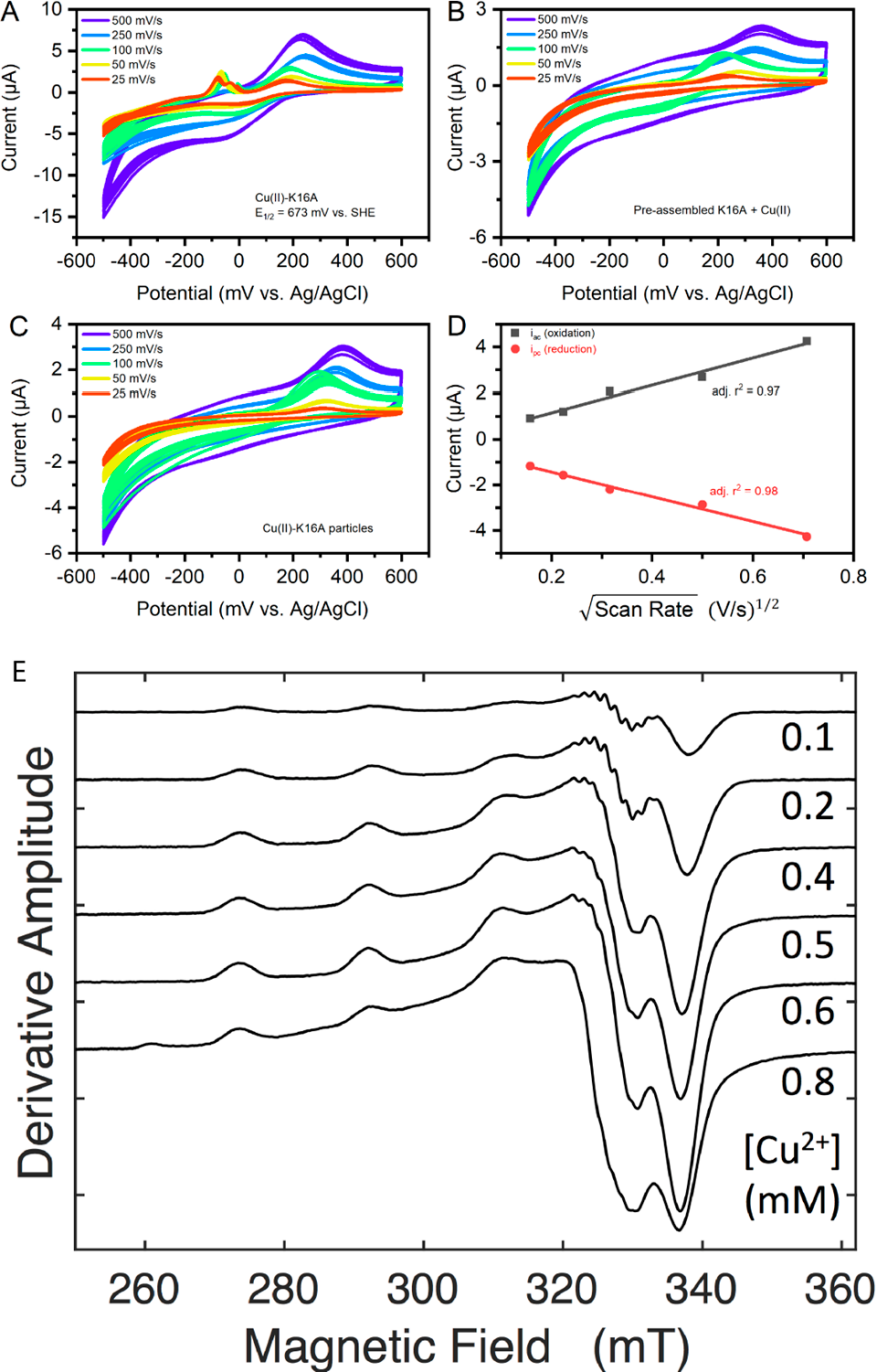
Cyclic voltammetric responses of coassembled Cu(II)-K16A (1:1 equiv) (A), preassembled K16A + 1 equiv of Cu(II) (B), initial Cu(II)-K16A coassembling particles (C), and the Cottrell-like plot of panel A’s data (D). CW-EPR spectra of Cu(II)-K16A for different [Cu(II)]/[K16A] ratios: 0.1 ≤ *x* ≤ 0.8. Each spectrum corresponds to the indicated Cu(II) concentration and 1.0 mM peptide. The absolute (unnormalized) amplitudes of the spectra are referenced to a common scale (a.u.) (E).

Titrating the Cu(II)-K16A coassemblies with stoichiometric reducing equivalents of ascorbate significantly reduces its 573 nm transition. When open to air, this transition gradually reintensifies over 16 h, consistent with Cu(I)-Aβ(1–40) that also recovers to 80% of its intensity following 19 h of air exposure.^33^ While some ribbon fragmentation appears by TEM in the Cu(II)-K16A coassemblies, overall “twisted ribbon membrane” morphology is preserved (Figure S3). Cyclic voltammograms (CV) for both Cu(II)-K16A coassemblies and the preassembled K16A bound with Cu(II) give broad Cu(II/I) waveshapes (Figure 2A), indicating slow and quasi-reversible electron transfer kinetics. The primary anodic and cathodic processes are visibly symmetric with reproducible peak currents over at least 7 cycles. Preassembled K16A titrated with Cu(II) however gives a voltammogram with no obvious redox couple across most scan rates (Figure 2B). A related amyloid congener, HAQKLVFFA-NH_2_ (H14A) that also preassembles into fibers with bis-*cis*-His Cu(II) ligation,^34^ displays an analogous irreversible anodic wave (Figure S4). Cu(II)-K16A’s nascent particle condensates (*t* < 1 h) are expected to have little long-range molecular order and show electrochemical responses (Figure 2C) most similar to preassembled K16A fibers + Cu(II) and preassembled H14A + Cu(II). Thus, the order generated by the Cu(II)-templated K16A assembly is distinct and stable to reversible electrochemical charging.

The *E*_*1/2*_ (673 mV vs SHE) of the Cu(II)-K16A coassembly exceeds the reduction potential in most mononuclear cupredoxins (like ETC’s plastocyanin) by over 2-fold.^35^ While sample–electrode adsorption dynamics may impact this measurement,^36^ amido ligands from backbone amides in Cu(II)-Aβ(1–16) give negative reduction potentials (e.g., −153 mV vs SHE).^37^ Furthermore, Cu(II)-K16A’s peak potential correlates linearly with log(V/s), exhibiting large peak-potential differences (Δ*E*_p_s) across all measured scan speeds (Figure S5). These observations are consistent with electron transfer kinetics being slow relative to the scan rate (Figure 2A). Overlapping chemically irreversible waves at −0.033 and −0.078 V appear at slower scan speeds (<0.050 V/s), which broaden and dampen at higher speeds. These features are assigned to the oxidation of Cu(I)-K16A by O_2_ to yield reactive oxygen species (ROS) such as H_2_O_2_ (Figure 2A), which has also been observed to evolve from other Cu(I)-Aβ complexes^31,38−42^ based on their presence in solutions of aquo-copper and absence when copper is substoichiometric relative to K16A_._ Trace amounts of uncomplexed Cu(II), however, could also activate oxygen at such overpotentials.^37^ To determine whether these Cu(II)-K16A ETCs can transfer their mediated electrons to a terminal acceptor, dissolved oxygen, an Amplex Red assay^43^ was used to fluorometrically report H_2_O_2_ evolution by Cu(II)-K16A following its reduction by ascorbate, which served as a reducing equivalent akin to NAD(P)H. Both the co- and preassembled Cu(I)-K16A produced less cumulative H_2_O_2_, 60% and 80%, respectively, relative to buffered Cu(II) and Cu(II)-K16A particles at 5.25 h (Figure S6). Lower ROS-turnover by Cu(I)-K16A relative to free Cu(I) recapitulates previous work on soluble Cu(I)-Aβ(1–16) and fibrillar Cu(I)^−^Aβ(1–42),^31^ consistent with the peptide limiting O_2_ accessibility to the bound Cu(I).

The coassembled Cu(II)-K16A’s current correlates linearly with the square root of the scan rate (Figure 2D), further supporting electrochemical quasi-reversibility. The apparent diffusion rate constant for nanoscale assemblies can be estimated from the CV data as 10^–13^–10^–14^ cm^2^/s (Table S2), orders of magnitude slower than plastocyanin (10^–7^ cm^2^/s)^44^ and hydrated Cu(II) (10^–6^ cm^2^/s).^45^ Slow-diffusing materials with linear Cottrell-like relationships have been observed in other bulky systems, including metalloporphyrin metal organic framework (MOF) thin films^46,47^ and ferrocene-embedded^48^ or Ru-(bpy)_3_-embedded polymers,^49^ where charge-hopping between metal centers facilitates electron flux between the electrode and the slow-diffusing bulk. Therefore, we interpret the coassembled Cu(II/I)-K16A’s broad, quasi-reversible redox couple to encompass multiple heterogeneous electron-hopping events between the bound Cu centers in the array. Unlike the MOF^46,47^ and embedded polymer systems,^48,49^ Cu(II)-K16A does not contain a porous network to accommodate shuttling counterions between electrode and analyte. Accordingly, the broad waveshapes likely stem from different orientations^36^ of the Cu(II)-K16A assemblies against the working electrode surface.

### Cu(II) Coordination in K16A Assembly

To interrogate copper ion coordination, electron paramagnetic resonance (EPR) spectra were collected on the Cu(II)-K16A coassemblies with metal/peptide ratios ranging from 0.1 to 2.0 (Figures 2E and S7). The EPR transitions show dominant features characteristic of Type II copper centers, specifically four copper (*I* = 3/2) hyperfine features centered around *g*_||_ that extend into a broad derivative-shaped feature in the higher-field *g*_⊥_ region, supporting axial symmetry at the copper centers. The super-hyperfine splitting at *g*_⊥_ is characteristic of coupling to multiple, near-equivalent ^14^N (*I* = 1) ligands from His-imidazole coordination. These type II Cu(II) sites are apparent in the EPR spectra from 0.1–0.6 mM Cu(II), with the amplitude diminishing with increasing Cu(II) concentrations. The appearance of characteristic Cu(II)-aquo features at [Cu(II)]/[K16A] = 0.8 (Figures 2 and S8) with a copper hyperfine peak at 262 mT and a second trough at *g*_⊥_ 327 mT), indicates that K16A binding sites saturate between 0.6 < [Cu(II)]/[K16A] < 0.8. This assignment is supported by the absence of bound-copper contributions and a linear increase in the free Cu(II)-aquo signal at [Cu(II)]/[K16A] > 0.8. EPR fitting details and acquisition parameters are in Figure S8.

Constraints on the peptide backbone packing were obtained with infrared analyses of K16A and Cu(II)-K16A assemblies. The sharp, red-shifted amide-I absorption bands at 1628 cm^–1^ (Figure S9) are diagnostic of extended coupling of H-bonded amides in β-sheets.^50,51^ Circular dichroism analyses further support that β-sheets compose these peptide membranes with positive ellipticity at 199 nm and broad negative ellipticity at 223 nm (Figure S10). These general β-sheet features are recapitulated in apo-K16A, as published previously.^20^ Powder X-ray diffraction (pXRD) analyses confirm the cross-β architectures with reflections at 4.7 and 9.9 Å for the orthogonal packing of strands and sheets respectively (Figure S11).^22,52^ To define the β-strand registry, isotopic enrichments were synthetically incorporated for {1-^13^C}-Ala16 and {^15^N}-Phe19, each placed sufficiently internal in the β-strand to reduce spin relaxation from the paramagnetic Cu(II) ions. Indeed, mature Cu(II)-K16A coassemblies formed with this enriched peptide give CP/MAS ^13^C NMR spectra dominated by a single sharp carbonyl transition at 172.6 ppm^20^ (Figure S12), consistent with this alanine reside residing in a β-sheet.^53^ Interstrand distances determined with ^13^C{^15^N} rotational-echo double-resonance (REDOR) NMR analyses^54,55^ place amide ^15^N atoms from adjacent H-bonded strands at 4.4 Å (*r*_1_) and 5.1 Å (*r*_2_) from the enriched carbonyl with a ^15^N–^13^C–^15^N dihedral angle of 153.0° (Figure S13). These constraints are consistent with β-strands arranged as either antiparallel in-register or out-of-register by two amino acids. In contrast, assembly of the ^13^C-enriched K16A peptide without Cu(II) gives fibers with strands arrayed as parallel in-register β-sheets by DQF-DRAWS in earlier work.^20^

Molecular modeling constrained by pXRD *d*-spacings and NMR distance assignments place the N-terminal histidine residues on the membrane leaflet surface as a 9.4 × 9.9 Å matrix when templated with Cu(II) (Figure S14). This matrix restricts the closest His-spacing to 9.4 Å, a distance that constrains the binding of the metal to strands bridging across the membrane leaflet surfaces. Atomic force microscopy (AFM) measurements (Figure S15) of the Cu(II)-K16A membrane thickness gave 5.29 ± 0.41 nm relative to 3.49 ± 0.25 nm for the apo-K16A fibers.^20^ Modeling a flat in-register K16A lattice gives a thickness of 3.2 nm for a single leaflet and 6.4 nm for a bilayered peptide membrane. The observed intermediate thickness for the Cu(II)-K16A membrane suggests a bilayer having each leaflet containing doubly out-of-register strands with the resulting flexible, overhanging histidine residues on the solvent-exposed faces that could be compressed during AFM measurements or Cu chelation. Consequently, this antiparallel, bilayered amyloid membrane doubles the metal-binding ligands at the leaflet interface, contributing to the degree of Cu(II) chelation in Cu(II)-K16A. This is distinct from full-length Cu(II)-Aβ, which positions the metal-binding domain only along the fibril’s solvent-exposed faces.^56,57^

**Figure 3:**
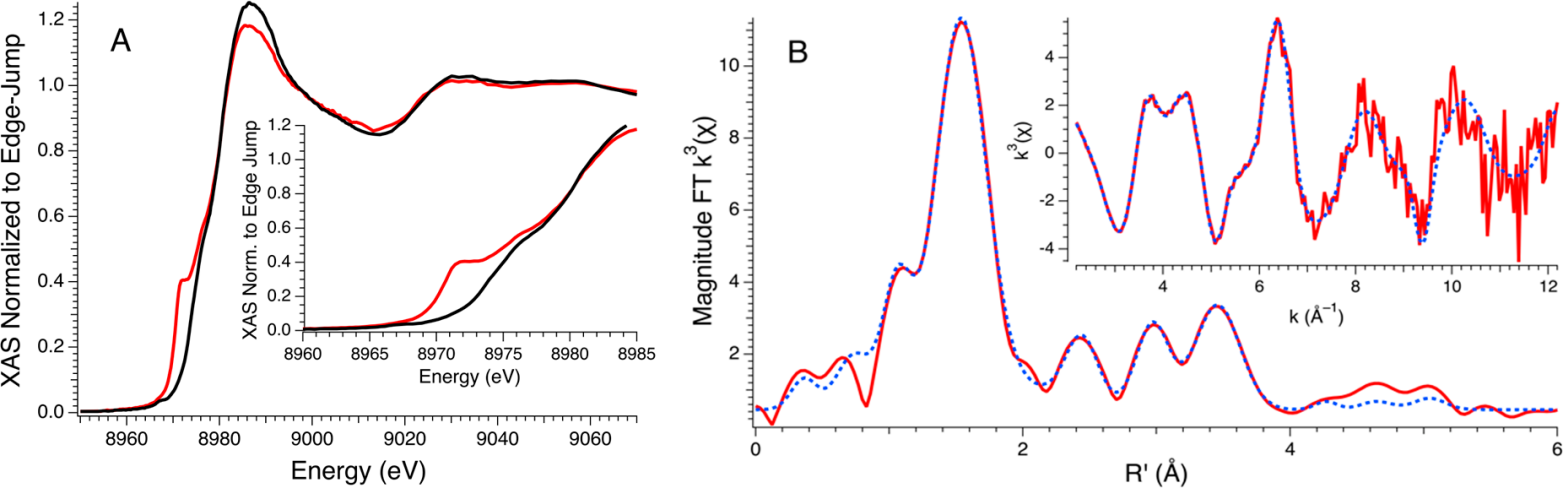
(A) XANES region of the Cu K-edge X-ray absorption spectrum for oxidized (black) and reduced (red) Cu–K16A assemblies with magnified (inset) pre-edge features. (B) *k*^3^-weighted EXAFS spectrum of Cu(II)-K16A (solid red) with reported bis-His fit (dashed blue) with *k*^3^-weighted magnitude Fourier transform of the EXAFS (inset). bis-His fit: *E*_*o*_ = 8984.3 eV Shell #1 Cu–O: *n* = 2 (restrained), *r* = 1.988(3) Å, σ^2^ = 0.0041(2) Å^2^; Shell #2 Cu-imidazole: *n* = 1 (restrained) *r* = 2.022(4) Å, σ^2^ = 0.0028(3) Å^2^, θ = 4(1)°, φ = 133(16)°; Shell #3 Cu-imidazole: *n* = 1 (restrained) *r* = 2.057(4) Å, σ^2^ = 0.0019(4) Å^2^, θ = 8(5)°, φ = 135(11)°; ε^2^ = 0.81. For definition of angles in fits, refer to Shearer and Soh.^58^

The Cu(II) coordination environment within the K16A assemblies was further analyzed with Cu *K*-edge X-ray absorption spectroscopy. To achieve adequate signal-to-noise, Cu(II)-K16A was prepared as a 6 mM solution with 0.2 equiv of Cu(II). These more concentrated preparations gave comparable twisted ribbon membrane structures by TEM and EPR line shape resembling the more diluted preparations (Figures S16 and S17). The XANES region of the Cu *K*-edge X-ray absorption spectrum of Cu(II)-K16A shows a weak pre-edge feature at 8,972.5(1) eV, corresponding to a nominal Cu(1s → 3d) transition and an additional shoulder in the edge at 8,976.6(3) eV, which is assigned as the Cu(1s → 4p + shakedown) transition (Figure 3). These two features support four-coordinate square-planar Cu(II).^59,60^ Analysis of the EXAFS region is also consistent with a four-coordinate Cu(II) site. Several statistically and chemically valid fits to the EXAFS data could be obtained with multiple Cu-imidazole coordination numbers (Table S3). Among these, we obtain a fit to the EXAFS data that contains a Cu–O single scattering pathway with two O scatterers at 1.99 Å, a Cu-imidazole multiple scattering pathway with a Cu–N distance of 2.02 Å, and an additional Cu-Im multiple scattering pathway with a Cu–N_δ^1^_ distance of 2.06 Å (Figure 3).

We note that titrating ascorbate to Cu(II)-K16A changes the XANES region of the copper K-edge X-ray absorption spectrum consistent with a 4-coordinate tetrahedral Cu(I)-center.^58^ The weak Cu(1s → 3d) transition is lost, while there is the appearance of a moderately intense Cu(1s → 4p) transition at 8,971.7(1) eV. A trigonal site assignment is less likely as the corresponding Cu(1s → 4p) transition would occur at higher energies (>8,980 eV) due to its higher symmetry. Overall, this structural change occurring at the Cu-center upon reduction^61^ could be responsible for the observed slow ET kinetics.

**Figure 4:**
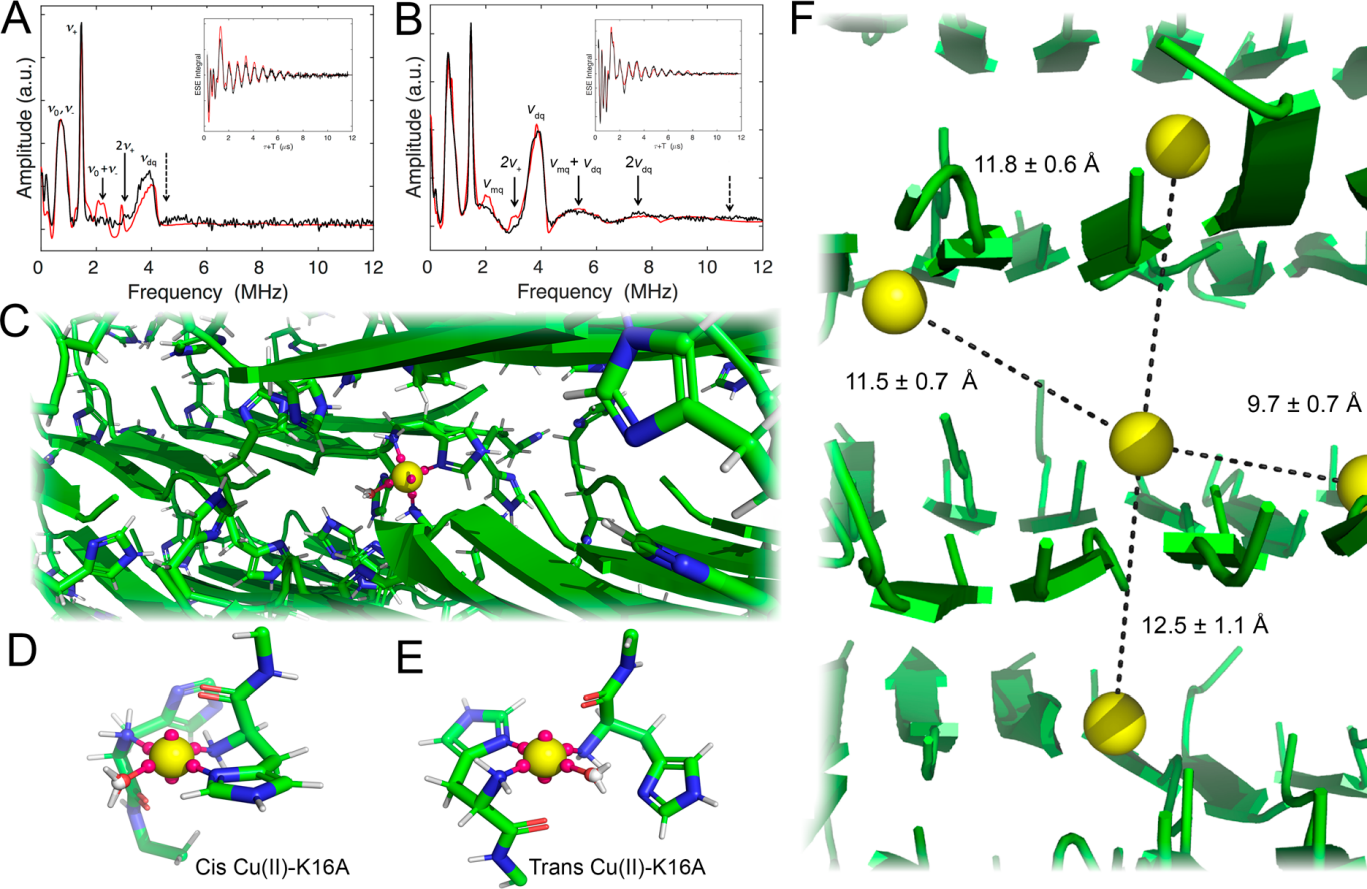
ESEEM frequency spectra (black lines) for dephasing times, τ, of 160 ns (A) and 310 ns (B) with respective FIDs (insets) and fits (red lines). Features arising from ^14^N nqi and super-hyperfine coupling with the unpaired electron spin on Cu(II) are described in the text and indicated by solid arrows. Positions of features that would be present for tris-histidine coordination are indicated by dashed arrows. Simulated mono-His Cu(II)-K16A assembly with ESEEM and XAFS restraints as a single site featuring a dummy copper atom (yellow sphere) with six ligand-localizing dummy charges (pink spheres). Nonhistidine side chains are hidden for clarity (C). Plausible, time-averaged coordination spheres were extracted from separate simulations featuring *cis* and *trans* positioned ((D) and (E), respectively) N-termini ligands consistent with the spectroscopic data. Simulated Cu(II)-K16A membrane lattice with five DCUs in adjacent laminates and strands with Cu(II)–Cu(II) distances shown. Side chains are hidden for clarity.

To further resolve the number of ligating histidines, three-pulse ESEEM spectroscopy^62^ detailed super-hyperfine coupling of the Cu(II) unpaired electron spin with the ^14^N nuclear spin of the remote nitrogen of the imidazole ligand (Figure 4).^63^ Pulse sequences delay times were selected to suppress the large ^1^H Larmor signal and to reveal combination features that arise from the ^14^N nuclear quadrupole interaction (nqi) (τ = 160 ns) and associated ^14^N super-hyperfine coupling (τ = 310 ns). The Fourier transform spectra show fundamental nqi frequencies (ν_0_, ν_–_, ν_+_) characteristic of the remote ^14^N of imidazole as well as a combination line at twice the fundamental value (2ν_+_), which indicates the presence of Cu(II) sites with two histidine imidazole ligands (Figure 4A). The double quantum (ν_dq_) feature also shows combination lines (2ν_dq_, ν_mq_ + ν_dq_) (Figure 4B). Higher-order combination features are not detected, indicating that each Cu(II) is coordinated by no more than two histidine residues.

The uniformity of the well-resolved nqi lines suggests that the histidine ligands have a common electronic structure and hydrogen bonding environments. Simulations of the ESEEM spectra reveal two dominant coordination environments in Cu(II)-K16A coassembled with 0.2 metal equivalents: discrete mono- and bis-His constituents existing in an approximate 4:1 ratio, respectively. The lineshapes of the 2ν_dq_ and ν_mq_ + ν_dq_ combination features also depend on the orientation of the histidine ligands as described by the corresponding ^14^N dipolar super-hyperfine principal axis systems (PAS).^34^ These axes have a defined relationship to the plane of the imidazole ring.^64,65^ ESEEM simulations^66^ that incorporate coupling of the electron spin with two ^14^N containing identical super-hyperfine and nqi parameters (Table S4) lead to Euler angles of [α β γ] between the super-hyperfine PAS corresponding to the bis-His constituent. The relationship between these two ^14^N PAS indicates that the PAS of the ligating histidine residues is rotated about Cu(II) through Euler angles of [−50°, −94°, −100°] ± 10°. Notably, immature 2 week incubated Cu(II)-K16A coassemblies show no detectable 2ν_dq_ combination line in the ESEEM Fourier transform.^34^ This initial mono-His ligation in Cu(II)-K16A’s oligomeric state highlights a transition to bis-*cis*-His coordination on maturation as documented in a previous kinetic assembly intermediate observed in two-step nucleation of other templated cross-β assemblies.^50^

### Molecular Models for the Cu(II)-K16A Coassemblies

A restrained 2 × 8 × 8 β-sheet lattice model was constructed using Maestro^67^ containing the experimentally defined amyloid *d*-spacings and the doubly out-of-register strand arrangement. This amyloid model was simulated for 1.2 ns,^68^ and the final frame of the trajectory was used as the scaffold for constructing the coordination sphere (Figure 4C). A dummy copper atom (DCU), parametrized by Liao et al.^69^ to recapitulate coordination geometry and verified in Aβ(1–16) and superoxide dismutase models, was placed within the peptide bilayer to facilitate square planar ligand placement. The DCU was placed between the fourth and fifth laminate and the third and fourth β strands of the model to bury the DCU from peripheral peptide strands, which were seen to dissociate from the membrane’s lattice over extended trajectories. For mono-His Cu(II)-K16A, the only reasonable ligand composition that achieves a theoretical *λ*_*max*_ within ±10 nm of the empirical value using Prenesti et al.’s^32^ ligand-spectrum correlation function is (Im)(ammino)_2_(hydroxo). Both *cis* and *trans* N-terminal ligand arrangements are consistent with the XAFS data and thus were tested in separate models. Histidine side chains and N-termini from each leaflet were rotated to within ∼2 Å of the DCU and affixed as bonds within the OPLS-AA force field^70^ between either His(N_δ^1^_) or the N-termini to 3 of the 4 individual dummy equatorial charges for a total 2 Å-ligand-Cu distance in accordance with the XAFS data. Upstream backbone starting coordinates were not altered.

The DCU-modified K16A scaffold was simulated for an additional 2 ns. Since pH was not defined explicitly within the solvent, a nonbonded TIP4P water molecule that freely associated with the fourth equatorial dummy site serves as a positional proxy for the hydroxo-ligand. No waters are associated with axial sites in either trajectory. Isolating the coordination sphere’s RMSD-stabilized frames identified average Cu-ligand bond length bounds within approximately 0.1 Å of the XAFS distances (Figure S18). Atomic coordinates within a 4 Å locus around the DCU in both the *cis* and *trans* cases were isolated and averaged (Figure 4D,E) to comprehensively represent Cu(II)-K16A’s dominant form(s).

A simpler DCU model was constructed to visualize Cu(II)-K16A’s less populous bis-His constituent. Instead of utilizing dummy charges, a −94° dihedral restraint between the bound imidazole planes and a 139.7° N_ε^2^_–Cu–N_ε^2^_ restraint were included in the topology to capture the ESEEM constraint. Final frame coordinates exported from this additional 2 ns trajectory were also used to simulate a pXRD pattern, which closely matched experimental reflections (Figure S19A). The RMSD of the peptide atoms within 4 Å of the DCU radius indicated that conformational stability was reached at 700 ps (Figure S19B). Cluster analysis recapitulates a 94 ± 14° dihedral angle between both histidyl moieties and the XAFS-commensurate DCU-His(N_δ^1^_) bond lengths. An unrestrained 91 ± 6° N_δ^1^_–Cu–N_δ^1^_ angle was revealed under these conditions (Figure S19C–F), indicating a bis-*cis*-His ligation akin to preassembled Cu(II)-H14A.^34^ The time-averaged coordination sphere and angle measurements are depicted in Figure S20. Further parametrizing the molecular dynamics simulations of the bis-*cis*-His model with the N-termini restrained to 2.0 Å from the DCU gives a (Im)_2_(ammino)_2_ geometry most compatible with the spectroscopic data (Figure S21). A representative DCU-K16A trajectory of a full membrane lattice plus DCU simulation can be found in Supplementary Video 1.

Another 2 ns Cu(II)-K16A simulation was performed with 4 more restrained DCUs positioned proximal to the nearest unoccupied histidine pairs flanking the initial metal center to further evaluate how K16A’s membrane lattice accommodated bound Cu. The cross-β *d*-spacings allowed for average DCU-DCU distances to be accommodated within 9.7–12.5 Å (Figure 4F). Such spacings are consistent with the CW-EPR data, where the absence of detectable dipolar broadening excludes Cu(II)–Cu(II) distances < ∼7 Å.^71^ The histidine N_δ^1^_ atoms were resolved within discrete regions, further resolving how K16A’s membrane lattice constrains the bound Cu(II) ions periodically (Supplementary Video 2). Biological precedence for serial electron transfer across analogous distances can be found in several natural electron transport chain proteins, like respiratory complex I^72^ or photosynthesis’s cytochrome *b*_6_f,^73^ whose numerous cofactors conduct multiple transfers across distances of 8–14 Å. In fact, the consensus for the maximum donor–acceptor distance allowed for electron transfer ranges from 14 to 20 Å.^74,75^

## Conclusions

These analyses provide insight into how a truncated amyloid peptide, K16A, experiences templated assembly by Cu(II) into ordered twisted ribbon membranes with mixed mono-His and bis-*cis*-His Cu(II) complexes arrayed within the peptide leaflet interface. Using electronic transitions, XAS, pulsed- and CW-EPR, and constrained molecular dynamics, we defined structural models for the detected Cu(II)-coordination modes within this assembly. Compared to Cu(II)-Aβ, Cu(II)-K16A features a dramatic rearrangement of the metal-binding sites with longer inter-Cu(II) distances and unexpected redox properties. The high density of histidines, N-termini, and passivating buffer salts^27^ should stabilize higher copper oxidation states, while the internal location of Cu(II)-centers is expected to preclude redox. Thus, we sought to understand how K16A’s cross-β membrane lattice informed the arrayed metal’s electronic properties and reactivity.

Entasis,^76,77^ where a protein’s scaffold increases the redox reactivity of metal ion cofactors by distorting orbital geometry, was first considered to stem from K16A’s specific cross-β scaffold. In addition to increasing the d-orbital splitting, such deviations from the idealized crystal field would also increase the reduction potential’s absolute value if the strained geometry approximated the resting product state. While Cu(II)-K16A coassemblies exhibit a rather blue-shifted *λ*_*max*_ and an oxidizing reduction potential, the ESEEM-restrained molecular dynamics predict an average bite angle of 91° ± 6°, which defines an unstrained *cis* geometry that refutes entasis-facilitated redox modulation. Additionally, sample adsorption on the electrode is known to shift peak potentials,^36^ as has been noted^38^ in previous reports featuring the neurotoxic Cu-Aβ assemblies.^78^ Since the reduction potentials of the ascorbate/dehydroascorbate and O_2_/H_2_O_2_ couples are 51^79^ and 295 mV vs SHE,^80^ respectively, we predict that the reduction potential of the Cu(II)-K16A coassemblies would fall within these bounds. Collectively, this micrometer-length, polynuclear metal-peptide membrane sustains iterative electron transfers across its ordered copper centers and between diffusible donors and acceptors. Further analyses of mediating redox to and from this engineered ETC using quinones, which serve as a naturally occurring redox shuttle in respiration,^3,81^ are currently underway.

**Figure 5:**
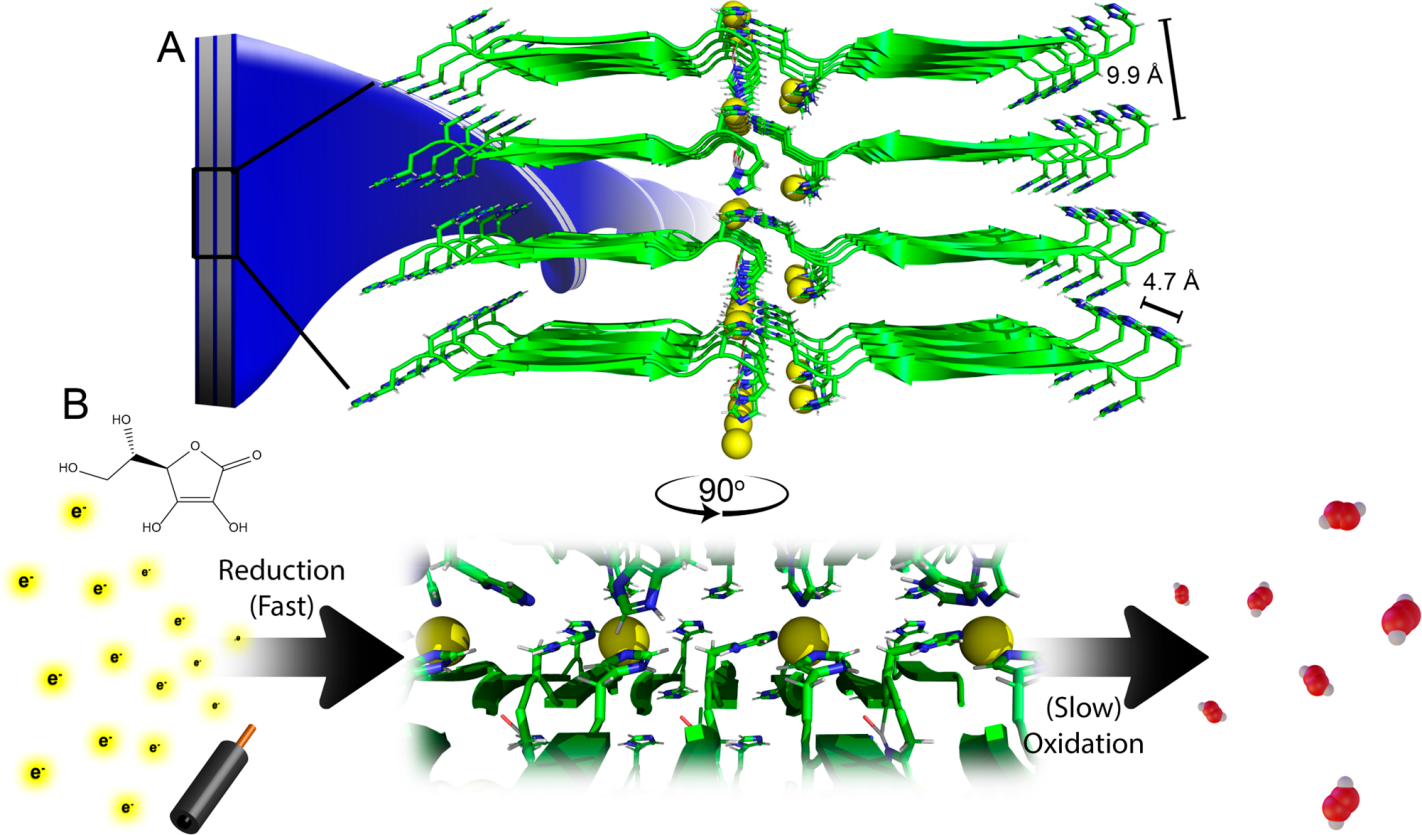
Cu(II)-K16A membrane cartoon incorporating pXRD *d*-spacings and their influence on the Cu(II/I) lattice (A, B) demonstrating how charge hopping might facilitate global “charging/discharging” of bound copper sites reduced through substrate or electrode and then cycled into ROS.

Beyond thermodynamics, Cu(II)-K16A’s redox cycle kinetics show that substrate-level reduction of Cu(II)-K16A coassemblies occurs within the mixing time, while oxidation by dissolved O_2_ proceeds more slowly. In the case of Cu-Aβ(1–40), this timing has been attributed to an uncommon O_2_-resistant^33,57,82−86^ linear *trans*-bis-His Cu(I) geometry and nonspecific interactions between the scaffold and dissolved O_2_. The latter property likely contributes to the relatively slow oxidation rate observed in the Amplex assay on Cu(II)-K16A coassembly’s rate, too, due to embedding of the copper ions within the peptide network. Notably, Cu(I)-K16A’s XANES spectrum indicates a tetrahedral geometry, which is not oxidation resistant.^87^ We then propose that the charge hopping inferred from CV facilitates the oxidation-resistant “wireless current” of these Cu(II) sites by external electron sources (Figure 5), so that oxidative pathways of a putative Cu(I) must compete with the transfer to proximal Cu(II) sites.

Assuming full site occupancy with a uniform barrier to electron transfer, a first-pass approximation of kinetics can be estimated with the Moser–Dutton ruler:^6^
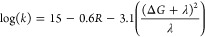
where *R* is the donor–acceptor distance (e.g., 9.7 Å), λ is the reorganization energy (assumed as 0.7 eV for intraprotein electron transfer^88^), and Δ*G* is the free energy (approximated as 0 eV, i.e., negligible thermodynamic driving force between identical sites). Here, *k*_ET_ = 1 × 10^7^ s^–1^ conservatively describes Cu(II)-K16A’s theoretical upper electron transfer rate between adjacent Cu(II) sites. Therefore, the electron-hopping rate between bound Cu(II) sites should be fast relative to that of Cu(I)-K16A’s oxidation. Likewise, fast Cu–Cu transfers relative to the reduction rate could further explain Cu(II)-K16A’s large Δ*E*_*p*_s yet unexpectedly linear signal-scan rate correlation.

This “electron bottlenecking”, achievable through designing a metal-binding peptide amphiphile that assembles iterating redox sites between protective bilayer leaflets, occurs through a dense matrix of Cu centers functioning as a robust ETC. Thus, we infer the limited access of the copper sites along the peptide membrane extremities gate-keeps electron flux between the assembly and outside oxidants, contributing to the robustness Cu(II)-K16A displays toward redox. Compared to the numerous lipids and 40+ discrete carriers required for electron transfer in photosynthesis^89^ and respiration^3^ combined, here we abridge and simplify a core process of natural ETCs to a two-item system of copper ions and peptide. To our knowledge, Cu(II)-K16A represents the first example of a self-assembling ETC comprising biologically compatible and nontoxic^18^ components, while achieving said charge hopping across distances on par with an organelle’s dimensions. Looking forward, introducing these arrays to bacterial cultures could signify a step toward bioorthogonally installing extracellular electron transfer to pre-existing metabolisms.

## Supporting Information Available

Peptide synthesis, purification, and assembly methods, spectroscopy including electronic spectra, circular dichroism, FTIR, and fluorimetry, EPR, solid-state NMR, XAS, ESEEM, as well cyclic voltammetry, powder X-ray diffraction, TEM, AFM, molecular modeling, MD, and ESEEM simulations (PDF)
Supplementary Video 1: Restrained molecular dynamics of Cu(II)-K16A(MP4)
Supplementary Video 2: Two-hundred superimposed frames of a 2 ns Cu(II)-K16A simulation with five dummy Cu(II) (MP4)
