# Supplementary material for: Engineering Synthetic Electron Transfer Chains from Metallopeptide Membranes
Source: Inorg Chem. 2023 Dec 21;63(6):2899–908. doi: 10.1021/acs.inorgchem.3c02861 (PMC10865380; doi:10.1021/acs.inorgchem.3c02861)
Supplement: Supplementary file 1 — ic3c02861_si_001.pdf [file ic3c02861_si_001.pdf]

Supporting Information

Engineering synthetic electron transfer chains from metallopeptide membranes

Anthony Sementilli[†], Rolando F. Rengifo[†], Wei Li*, Andrew M. Stewart*, Katie L. Stewart*, Umar Twahir*, Youngsun Kim†, Jipeng Yue[†], Anil K. Mehta[†], Jason Shearer[◆‡], Kurt Warncke*[‡], David G. Lynn[†§‡]

Departments of Chemistry†, Biology§, and Physics*, Emory University, Atlanta, GA 30322.
Department of Chemistry◆, Trinity University, San Antonio, TX 78212

Synthesis and purification of K16A

Peptides were synthesized on a CEM Corporation microwave peptide synthesizer (Matthews, NC, USA) using standard Fmoc chemistry. The solid support for peptide synthesis was an Fmoc rink-amide resin (AnaSpec, Inc., Fremont, CA, USA) with a typical substitution range of 0.4-0.6 meq./g. Resin was pre-swollen in minimal dimethylformamide (DMF) for 15 min. Microwave assisted Fmoc deprotection was completed using 20% piperidine in DMF at 45-55°C for 180 sec followed by three DMF flushes. Each coupling step was performed using 0.1 M Fmoc-protected amino acid and activated with 0.1 M 2-(1H-Benzotriazole-1-yl)-1,1,3,3-tetramethyluronium hexafluorophosphate (HBTU) and 0.2 M N,N-diisopropylethylamine (DIEA) in DMF. Microwave coupling temperatures were maintained within 75-82 °C for 300 s then rinsed with DMF 3x. Histidines were doubly coupled at 50 °C with 11 W power instead of 8 W. After cleavage with trifluoroacetic acid/thioanisole/EDT/anisole, 95/5/3/2 v/v/v/v, crude peptide was extracted 3x in -20 ^{o}C ether and vacuum desiccated.

Peptides were purified using a Waters HPLC with a XSELECT™ CSH™ Prep C18 column (Waters, Milford, MA, USA). K16A eluted in 29% MeCN with a flow rate set at 60 mL/min, run at room temperature with a reverse-phase gradient of H_2O/MeCN. All solvents contain 0.1% TFA. Gradient method was optimized to allow for a minimum of 10 min before sample peak elution and a minimum of 5 min after peak collection before column re-equilibration. Gradient ramp rate = 1%/min. Fraction quality was assessed by MALDI-TOF on an Applied Biosystem 4700 Proteomics analyzer with a 355 nm ND:Yag laser operating at 200 Hz (Foster City, CA, USA). The matrix used to facilitate analyte desorption was α-cyano-4-hydroxycinnamic. (HHQALVFFA-NH_2, 1068.228 $(M+H^+)$). Confirmed product fractions were concentrated in vacuo before lyophilizing in a FreeZone Plus 12 Liter Cascade, Labconco, freeze dryer system (Kansas City, MO, USA). Lyophilized peptides were stored at room temperature inside a vacuum desiccator.

Peptide Assembly

K16A assemblies and buffers were prepared using ASTM Type II metal-free water (EMD Chemicals Inc., Gibbstown, NJ, USA) with 25 mM MES, pH 5.6.

<u>K16A fibrils</u>: K16A peptide was dissolved at 2x the desired concentration in metal-free water and vortexed for 5 sec. This 2x peptide stock was diluted to full volume with 50 mM MES buffer, pH

5.6, accompanying another vortex period. Peptide solutions assembled at room temperature for >1 week.

Pre-assembled K16A-Cu(II) fibrils: Mature K16A fibrils were titrated with a small volume of 100 mM $CuCl_2$ solution to the desired metal equivalent.

Co-assembled Cu(II)-K16A nanoribbons: a 2x peptide stock was prepared as described previously, except with the 100 mM Cu(II) stock addition occurring directly after diluting to 1x with 50 mM MES, along with a third vortex period. Note, the volume of water used to prepare the 2x stock was reduced to accommodate the final addition of Cu(II) stock. Peptide solutions assembled at room temperature for >2 months.

Visible Absorbance Spectroscopy

Scatter-free absorbance spectra of peptide-metal assemblies were measured with an RSM 1000 at Olis, Inc. (Bogart, GA). Samples were diluted 2x with buffer to a final volume of 3.2 mL in a 9 mL DeSa Suspension Presentation Cavity. Spectra were collected with an 850-400 nm grating, 2.25 nm data interval, and a 180 nm/min scan rate.

Cyclic Voltammetry

Cyclic voltammetry was performed at room temperature using a WaveDriver potentiostat (Pine Research Instrumentation, Durham, NC, USA). Experiments used a standard three-electrode arrangement and a microvolume cell. Electrodes used were a 1 M Ag/AgCl reference electrode (0.228 V vs SHE), a Pt wire counter electrode, and a glassy carbon working electrode (CH Instruments Inc. Electrochemical Instrumentation, Austin, TX, USA). Buffered sample (2 mM peptide + 2 mM $CuCl_2$) was taken in 75 uL aliquots and diluted 2x with 25 mM MES, pH 5.6 + 100 mM KCl. The supporting electrolyte was 25 mM MES, pH 5.6 + 50 mM KCl. Cyclic voltammetry was conducted in the potential range of -0.500 to 0.600 V vs. Ag/AgCl at scan rates of 0.025, 0.050, 0.100, 0.250 and 0.500 (V/s). The analyte's open circuit potential was defined as the starting sweep potential, and all runs were cycled starting in the positive potential direction. Before each measurement, the electrolyte was degassed with N_2 for ~1 min. The working electrode was polished before use and in-between runs with an alumina slurry. A buffer measurement between each run at a scan rate of 0.100 V/s verified each experiment was performed with a clean working electrode with no detectable carryover signal/drift. Data was processed in AfterMath. Signal intensities were baseline-corrected in the software using its built-in free-sloped correction function, using a tangent of the preceding wave to define the baseline current. To convert Ag/AgCl-referenced potentials to SHE-referenced reduction potentials at any given pH at 25 °C: $E_{(SHE)} = E_{Ag/AgCl} + (0.059 \text{ x pH}) + E^{o}_{Ag/AgCl}$, where pH = 5.6 and $E_{Ag/AgCl}$ = 0.228 V. The first cycle from each run was discarded.

Diffusion constants were calculated from the Randles-Sevcik equation modified for electrochemically quasi-reversible systems at room temperature: $i^{quasi} = 2.62\ X\ 10^{5} FAcn^{\frac{3}{2}}\sqrt{Dv}$, where i^{quasi} = forward voltammetric current (A), F = Faraday's Constant (96,485 C/mol), A = electrode area (0.071 cm^2), c = concentration (0.0008 M), n = number of electrons (1), v = scan rate, and D = diffusion coefficient (cm^2/s)[2].

Circular Dichroism Spectroscopy (CD)

CD spectra were recorded using the Jasco-810 CD spectropolarimeter at room temperature. Spectra between 260 nm and 180 nm were collected with a 0.1 mm path length cell, with a step size of 0.2 mm and a speed of 50 nm/s. All spectra were recorded in triplicate and averaged. Ellipticity (mdeg) was converted to mean residue molar ellipticity (MRME, [θ], deg•cm^2•dmol^{-1}) by [θ] = θ/(10•n•C•l), where 'n' is the number of backbone amide bonds per peptide, 'C' is the molar concentration (mol/L) and '1' is the cell path in cm.

Fourier Transform Infrared Spectroscopy (FT-IR)

All FT-IR spectra were recorded using a Jasco FT-IR 4100 (Easton, MD, USA). Aliquots (10 μL) of peptide solution were fan-dried onto a Pike GaldiATR (Madison, WI, USA) diamond at room temperature and averaging 1,068 scans with 2 cm^{-1} resolution. Background spectra were subtracted from each sample.

Fluorimetry

H_2O_2 production was measured using a commercially available Amplex Red assay kit (Sigma) in stickered 96-well, black-walled, optical glass-bottom plates (Greiner Bio-one). Samples and standards were prepared as described by the kit's instructions with no procedural modifications. Samples were diluted 20x in buffer and reaction mixture prior to measurement. Cu(I)-mediated ROS production was initiated with freshly prepared 0.45 eq. sodium ascorbate (0.9 reducing eq.) prior to mixing with the kit reaction mixture. Sacrificial reductant was intentionally supplied as the limiting reagent as it triggers H_2O_2 production in the kit's master mix through the activity of the included horseradish peroxidase. All experiments were performed in a Synergy Mx plate reader (BioTek) with 300 μL sample volume maintained across all wells. The scan rate was set to 1 read/10 min with a 7 s shake period prior to every measurement.

Powder X-Ray Diffraction (pXRD)

Assembled K16A fibers and ribbons were collected by centrifugation using an Eppendorf centrifuge 5810 R (Mississauga, Ontario, CAN) at 4000 rpm held at 4 °C to prevent heating and melting the sample and to remove free monomer and small heterogeneities from the assembly mixture. The pelleted sample was decanted, resuspended in metal-free water, and re-pelleted 3x to remove buffer salts as well. The washed pellet was frozen and lyophilized to yield a dry powder for pXRD. The powder spectra were obtained using APEXII diffractometer with a Cu X-ray source, provided through Emory's X-Ray Crystallography Center.

Transmission Electron Microscopy (TEM)

10 μL K16A assembly aliquots were drop cast onto CF200-Cu Carbon Film 200 Mesh Copper grids (Electron Microscopy Sciences, Hatfield, PA). Peptide assemblies were settled on the grid surface for 1 minute. Excess solution was wicked away using a kimwipe. Immediately following, 10 μL of a 1.5% uranyl acetate stain was applied for 20 s and similarly wicked way. TEM grids

were vacuum desiccated overnight before imaging. Electron micrographs were imaged with a Hitachi HT-7700 Microscope at 80 kV at the Robert P. Apkarian Integrated Electron Microscopy Core.

Image measurements were performed using ImageJ, which were fit to Gaussian functions in Origin 2018b.

Atomic Force Microscopy

Samples were diluted 100-200x to ensure sample dispersion, 45 µL aliquots were drop cast onto silicon chip for 2 min (Ted Pella Inc., Redding, CA, USA). Excess solution was removed via micropipette.

Tapping mode analysis on a Veeco Dimension 5000 atomic force microscope with a full acoustic enclosure was employed using ultra-sharp non-contact silicon cantilevers with typical frequencies ranging from 150 kHz for the largest tip to 315 kHz for the smallest tip (NSC35/AIBS, MikroMasch, Watsonville, CA, USA). Samples were imaged simultaneously in topography and phase modes, with a filter of 0.2 Hz, a scan clock of 0.1667 ms, and a reference of at 70% of the maximum amplitude. The images were analyzed in Gwyddion.[3]

Electron Paramagentic Resonance (EPR)

EPR simulations of spectra obtained for the range of Cu(II)–assembly binding, at [Cu(II)]/[K16A] ratios of 0.1 – 0.6, indicate contributions from three dominant components (**Fig. 3**, **Table S2**). The [Cu(II)]/[K16A] = 0.1 spectrum cannot be simulated by using a single set (single Cu(II) species) of EPR simulation parameters (electron *g* and strain-broadening, Cu hyperfine, ^{14}N superhyperfine), because the amplitude of the simulated ^{14}N superhyperfine multiplet, associated with the requisite Cu(II) *g*- and hyperfine parameters, exceeds the experimental amplitude by >5-fold. This introduces a critical constraint on the model for the different Cu(II) site contributions. The overall Cu lineshape is preserved, and the ^{14}N superhyperfine amplitude attenuated, if a second Cu(II) site (Component 2) with enhanced strain-broadening is present, relative to the Cu(II) site (Component 1) with less strain-broadening, that expresses the ^{14}N superhyperfine features. At [Cu(II)]/[K16A] = 0.1, Components 1 and 2 are present in proportions of 80 and 20%, respectively, and there are no additional significant components (**Fig. S7**).

Components 1 and 2 are present in the spectrum at [Cu(II)]/[K16A] = 0.2 at altered proportions of 13 and 72%. A small deviation of the sum of Components 1 and 2 from the observed spectrum in the g_{perp} region, that extends into the $g_{||}$ region, is accounted for by a Component 3 (15%), which is characterized by a broad derivative feature and absence of distinct Cu hyperfine features. The decline in ^{14}N superhyperfine amplitude at [Cu(II)]/[K16A] = 0.4 is accounted for by a relative decrease in the contribution of Component 2 (7%). Component 1 also decreases (53%) and is compensated by a rise in Component 3 (40%). At [Cu(II)]/[K16A] = 0.6, which approaches the saturation of Cu(II) binding sites, Components 1, 2, and 3 reach proportions of 6, 47 and 47%, respectively. These proportions appear to persist at [Cu(II)]/[K16A] ≥ 0.8, in the range where the dominant aqueous Cu(II) spectrum precludes quantification of bound Cu(II) line shape contributions (**Fig. 2E**).

Solid-State Nuclear Magnetic Resonance

HHQ[1-^{13}C]ALV[^{15}N]FFA-NH_2 (enriched K16A) assemblies were pelleted, decanted, and washed with metal-free water 3x at 4°C for 90 min using an Eppendorf centrifuge 5810 R (Mississauga, Ontario, CAN) at 4,000 to remove any unassembled monomer and buffer. The washed pellet was lyophilized to a powder. TEM confirmed assembly persistence following lyophilization. The NMR samples were packed into 4 mm solid-state NMR rotors and centered using boron nitride spacers. Rotational-Echo DOuble-Resonance (REDOR)[4] spectra were collected as described previously[5]. In dephasing the carbonyl carbon of HHQ[1-^{13}C]ALV[^{15}N]FFA-NH_2 nanoribbons, the distance of the H-bonded ^{15}N from the adjacent peptide was set to 4.93 Å (r_1) and the distance to the non-H-bonded ^{15}N was set to 6.0 Å (r_2). The angle between the two ^{13}C-^{15}N internuclear vectors was set to 114.7°. The experimental data were fit to a linear combination of 3-spin (one ^{13}C and two ^{15}N's) and ^{13}C{^{15}N}REDOR curve corresponding to the ^{13}C-^{15}N distances sufficient to fit the experimental data points using the Non-Linear Fit routine in Mathematica.

X-ray Absorption Spectroscopy (XAS)

XAS Spectra were collected at Argonne National Laboratory (ANL) on beamline 20-B in fluorescence excitation mode with a 1 x 1 mm X-ray beam 10% defocused at 9.5 KeV (harmonic cutoff = 13.2 KeV). An insertion device beamline was not utilized to minimize potential beam damage.

The data were collected at 72 K, which was maintained by a liquid nitrogen cryostat (< 10 mTorr) with a sample-to-edge distance of 25 mm and a detector-cryostat distance of < 45 mm. A 12-element Ge detector with 2 us peaking time and 0.55 s integration time was used.

All samples were pelleted and resuspended in buffer to 80% original volume to remove potential peptide monomer-Cu(II) complexes. Immediately before freezing, samples were diluted back to the original volume with glycerol and mixed thoroughly to prevent Bragg scattering by water ice crystallites. Reduced samples were prepared in a portable glovebag (Sigma) that was purged 5x with argon.

Samples were aliquoted into a 3D-printed sample holder supplied by the beamline. The sample holder had two sample chambers stacked vertically, each with a volume of 350 uL and an 18 x 5 mm window area. The window material consisted of kapton film that was secured via an aluminum frame and steel screws. The entire cuvette was covered with a piece of tin sheeting with slits cut in to expose the windows while preventing excitation of the adventitious copper detected in the screws.

To minimize beam damage, sample was aliquoted in both chambers in duplicate with twenty scan sites that alternated across both sample chambers. Each scan collected 467 data points over 13.42 min between 8,830 and 9,953 eV.

All spectra were calibrated to standard copper foil data[6] in Athena (Demeter)[7] and individually inspected for photodamage. Satisfactory replicate scans were aligned, deglitched, and merged.

XAS data was fit in a custom IgorPro routine[8] as previously described in Shearer and Soh, 2007[9].

Molecular Modeling and Molecular Dynamics (MD)

Peptide assembly models were generated using Maestro 11.4 (Schrodinger Suite).[10] K16A peptides were extended as antiparallel β sheets and arranged into a bilayered lattice of 8 β sheet chains consisting of 8 peptides each for a total of 128 peptides. Peptides were spaced according to the hydrogen-bonding and lamination distances reported by pXRD, and the peptide registry according to the 13C{15N} REDOR fit.

Unrestrained MD was performed on the peptide lattice using GROMACS v2018.3[11] in a centered cubic box of TIP4P[12] water with periodic boundary conditions and a 1.0 nm protein-box distance using the OPLS-AA[13] force field. Short-range Van Der Waals and electrostatics were cutoff at 1.0 nm each using the Verlet scheme[14]. The system was neutralized with Na^+ and Cl^-. The system was energy minimized for 1,000 steps using a steepest descent algorithm then equilibrated via 100 ps NVT and 100 ps NPT ensembles. Average temperature was kept at 300 K using a Berendsen thermostat[15] with a 1.0 ps time constant, and average pressure was kept at 1 bar using a Parrinello-Rahman barostat[16] with a 2.0 ps time constant. MD was performed until the aligned protein's RMSD converged at approximately 1.2 ns. Bond distances were constrained with the LINCS algorithm[17] with a 2-fs integration time step.

The final frame's coordinates of the apo-peptide lattice were simulated with a dummy copper (DCU) to estimate the coordination sphere geometry. The DCU was constructed from a centroid placed within the bilayer and between the 4th and 5th β sheets to minimize the effects of bulk solvent exposure and peripheral β sheet peeling. The DCU was repositioned into ligation proximity using Chimera. To minimize experimenter bias, only the Cα-Cβ and Cβ-Cγ bond torsions of the N-terminal HISE were adjusted. The DCU-containing models were simulated identically to the apo-lattice, until the RMSD of an aligned, water-excluded 4 Å radius of DCU visibly plateaued.

The exported coordinates and the OPLS-AA force field was modified to include the experimental parameters from XAFS and ESEEM and simulated for 2 ns. RMSD typically converged in > 1 ns.

MD trajectory analysis (trajectory RMSD, bond length measurements, *etc.*) was performed using VMD.[18] Trajectory visualization/rendering and model raytracing was performed using PyMOL.[19]

A .pdb of the final frame was used to simulate a pXRD pattern using CRYSOL (ATSAS suite).[20] Default simulation parameters were used except for the order of harmonics and the order of the Fibonacci grid, which were maximized. The simulated pattern was normalized in Origin 2018b.

Electron spin-echo envelope modulation (ESEEM) spectroscopy.

ESEEM data were collected at 6 K on a home-built pulsed-EPR spectrometer[21] by using the three-pulse (π/2- τ-π/2-T-π/2- τ-echo) microwave pulse sequence with pulse-swapping.[22] The τ value was

selected from a scan of the τ values, corresponding to the relation, $\tau = \frac{n}{\nu_{^1H}}$, where n = 1, 2, 3, … and $\nu_{^1H}$ is the free precession frequency of the ^{1}H nucleus at the prevailing external magnetic field. These τ values correspond to τ-suppression [23] of the free ^{1}H contribution to the ESEEM. The τ values of 160 ns (n = 2) and 310 ns (n = 4) were chosen to emphasize the low-frequency ^{14}N nuclear quadrupole features and their combination lines, and the ^{14}N double quantum line (ν_{dq}) and its corresponding combination feature ($2\nu_{dq}$), respectively. ESEEM was cosine Fourier transformed to generate the ESEEM frequency spectra. Modulation in the dead time portion of the ESEEM waveform was reconstructed by inverse-Fourier transformation of prominent features in the initial Fourier transform.[24] All data processing was performed by using laboratory-written MATLAB (Mathworks, Natick, MA) programs running on local computers.

Electron spin-echo envelope modulation (ESEEM) simulations.

Numerical simulations of the ESEEM were performed by using the OPTESIM ESEEM simulation and analysis software suite.[25] Simulation of ESEEM that arises from the coupling of the remote ^{14}N of imidazole ligands to the unpaired electron spin on Cu(II) has been described in detail.[26] Briefly, the coupled electron–single ^{14}N system is parameterized by using the following eight adjustable parameters: A_{xx}, A_{yy}, A_{zz} (electron-^{14}N superhyperfine coupling constants), e^2qQ/h and η (^{14}N nuclear quadrupole coupling constant and asymmetry parameter), and α_Q, β_Q, γ_Q (Euler angles that relate the principal axes of the superhyperfine and nuclear quadrupole tensors). For simulation of the Cu(II)-bis-imidazole complexes, these eight parameters are assumed to be common to the two imidazole ^{14}N. For the bis-imidazole complex, α_A, β_A, and γ_A (Euler angles that relate the two ^{14}N superhyperfine dipolar tensor axes) are additional adjustable parameters. The simultaneous confidence region for each adjustable parameter, at a specific confidence level (here, 99%), was calculated, as described.[25] **Table S4** presents the values of the simulation parameters.

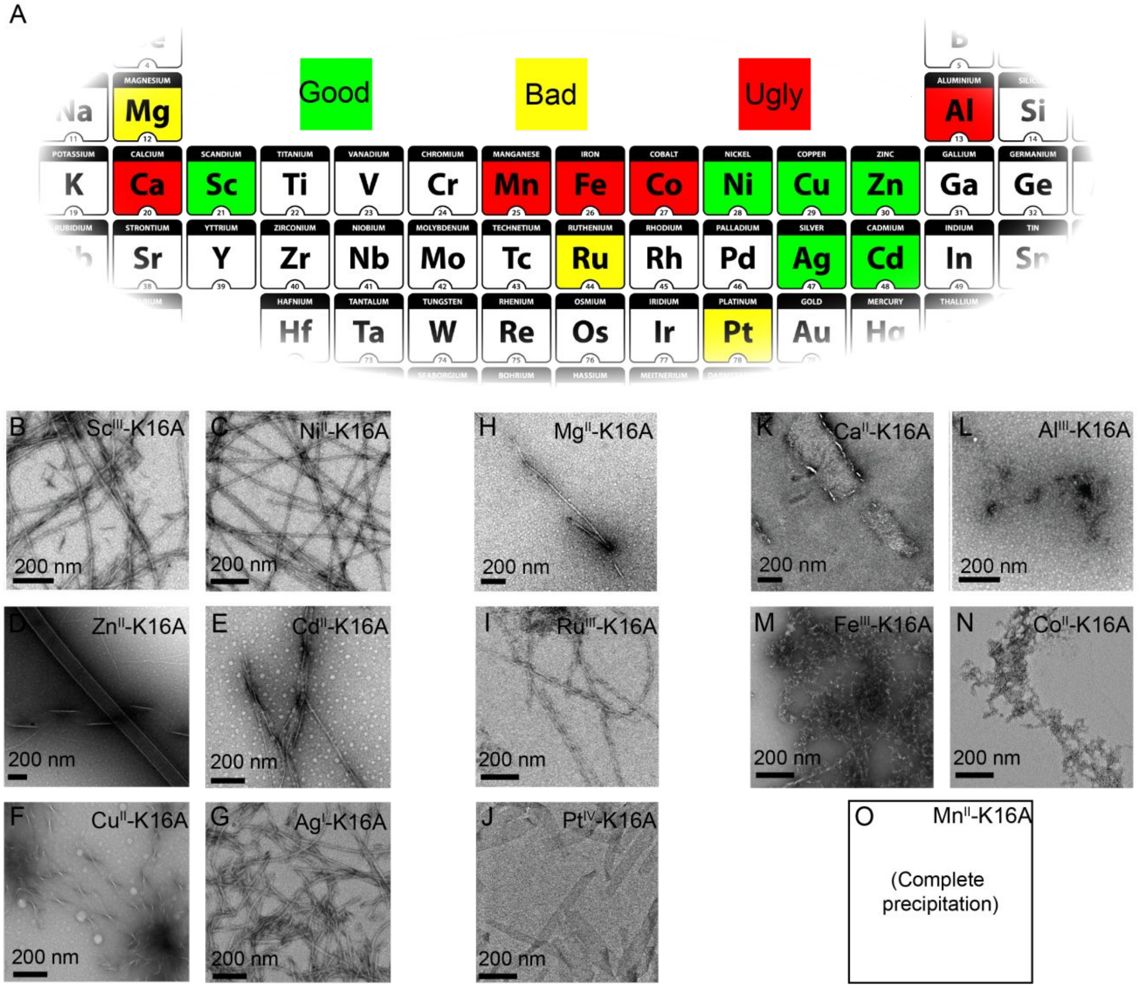

Figure S1. Periodic table-view screening of attempted metal-K16A assemblies to date, displaying several morphologies such as fibers (*e.g.* Ni(II)), nanotubes (*e.g.* Zn(II)), helical ribbons (*e.g.* Zn(II), too), twisted ribbons (*e.g.* Cu(II)), belts (*e.g.* Pt(IV)), aggregate (*e.g.* Fe(III)). (**A**). Assemblies prepared with green-labeled elements readily yield characterizable K16A assemblies by TEM. Elements labeled yellow produce mixtures of assembly and amorphous aggregate. Red elements form less structured associations with K16A. Representative TEMs of metal-K16A assemblies are shown in panels **B-O**. All assemblies were prepared as 1:1 co-assemblies in 25 mM MES, pH 5.6. Chloride salts were used as the metal ion source, except for Al(III) and Ag(I), which were introduced as nitrate salts. The periodic table was adapted from freestock.ca.

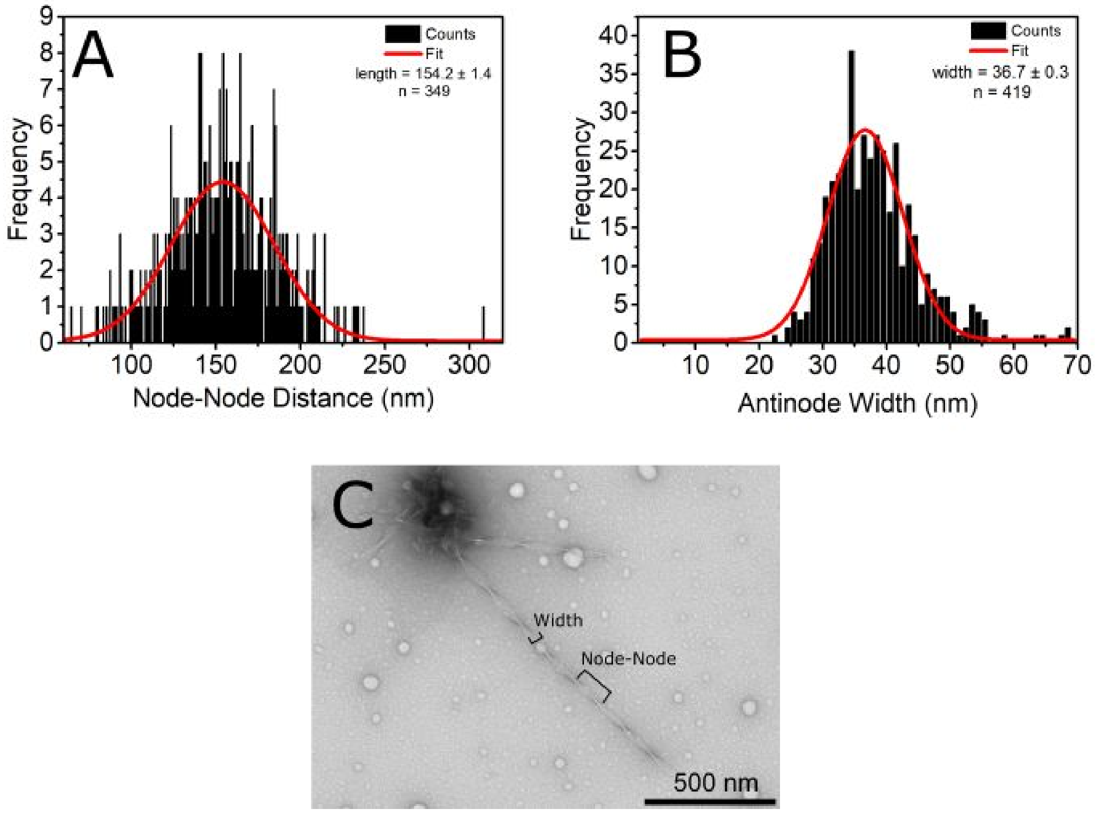

Figure S2. TEM statistics on Cu(II)-K16A's internode distances (A) and widths (B). Representative TEM displaying sample measurements (C).

Ligand Composition	Estimated λ_{max} (± 10 nm)
**$(Im)_2(H_2O)_2$**	692
**$(Im)_3(H_2O)$**	634
**$(Im)_3$(ammino)**	625
**(Im)(ammino)$(hydroxo)_2$**	604
**$(Im)_2$(ammino)(hydroxo)**	590
**(Im)$(ammino)_2$(hydroxo)**	582
**$(Im)_2(ammino)_2$**	570
**Experimental**	
**Cu(II)-K16A (co-assembled)**	573
**Cu(II)-K16A (pre-assembled)**	600

Table S1. Predicted λ_{max} values of for chemically reasonable ligand compositions calculated according to Prenesti et al.[1] along with experimentally measured values for Cu(II)-K16A assemblies.

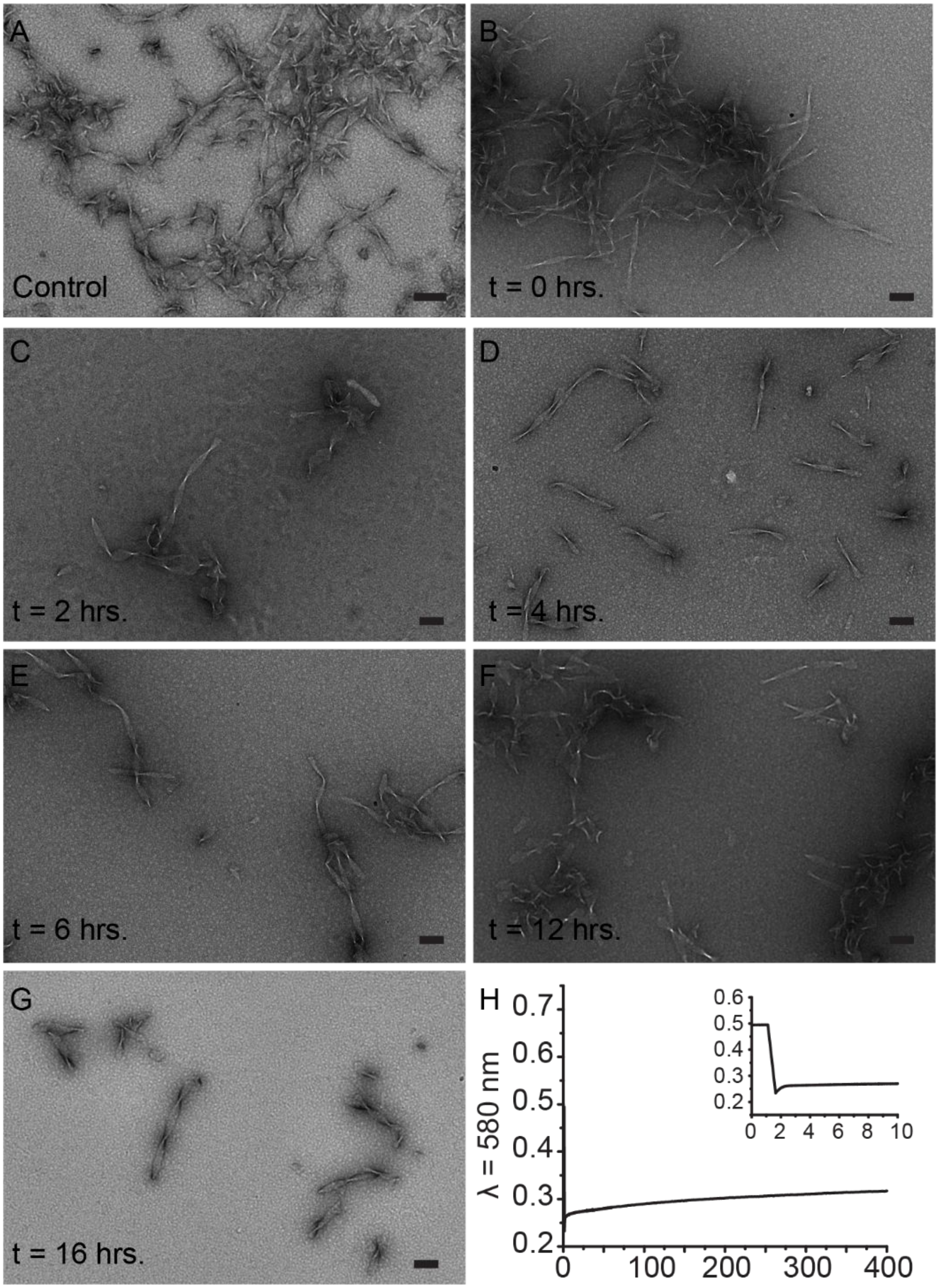

Figure S3. Cu(II)-K16A ribbons reduced via stochiometric ascorbate. (**A**) 2 mM ribbons before ascorbate addition. (**B-G**) Ribbon morphology persists following ascorbate addition. (**H**) Absorption kinetics in hrs following ascorbate addition to mature Cu(II)-K16A's λ_{max}.

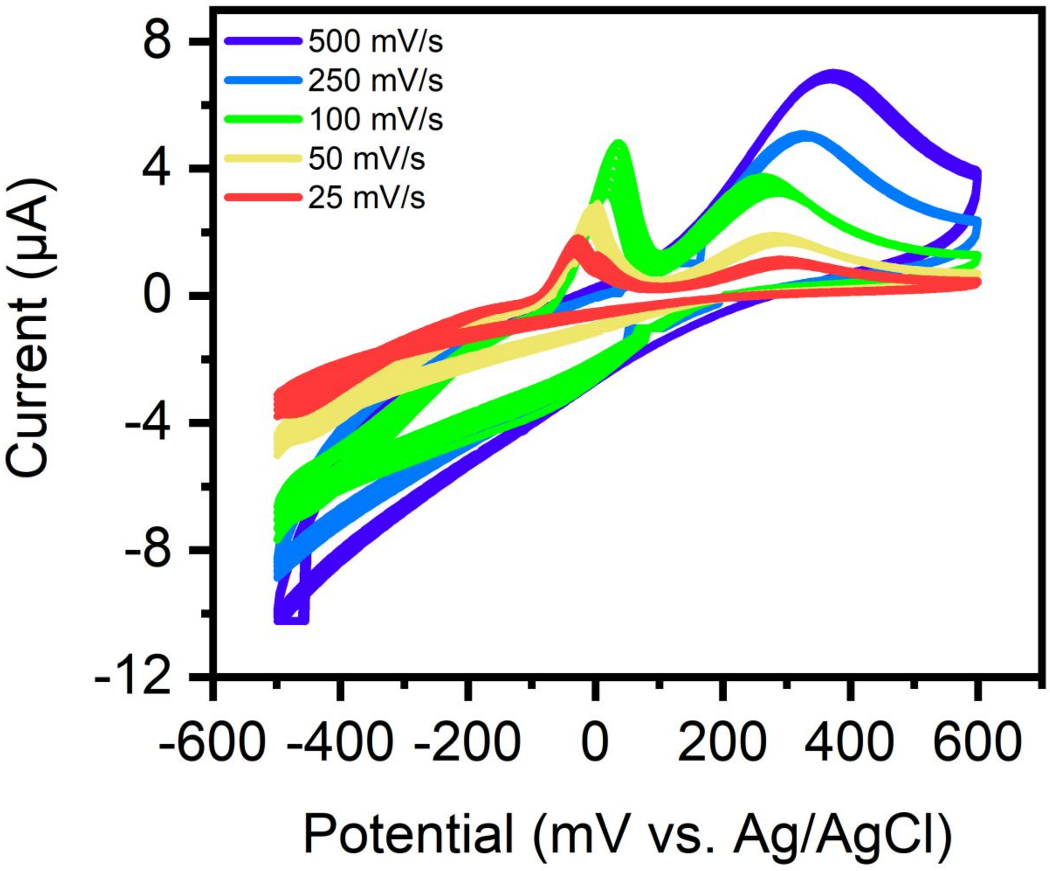

Figure S4. Cyclic voltammogram of pre-assembled Cu(II)-H14A in 25 mM HEPES + 10 mM NaCl at pH 7.2.

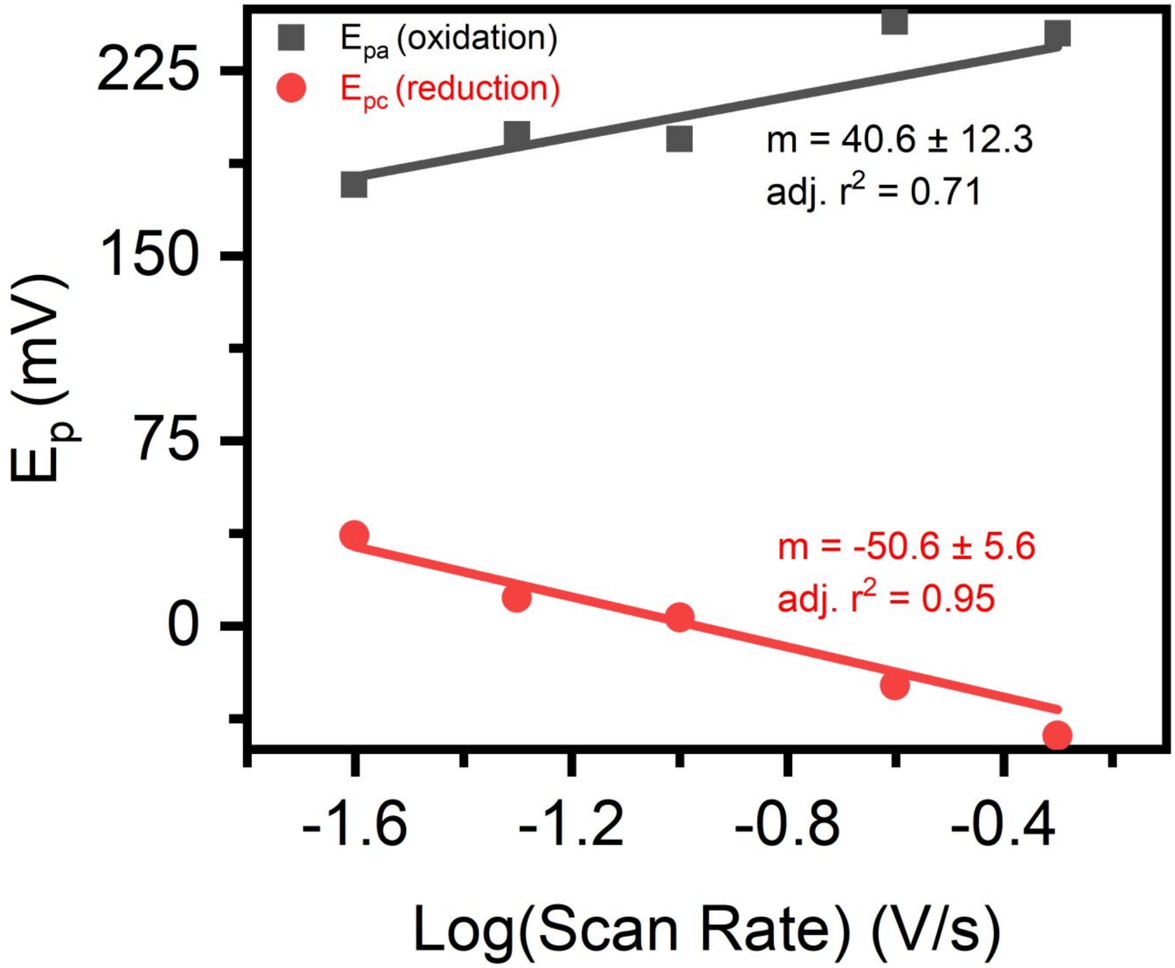

Figure S5. Cu(II)-K16A's peak potential vs. log(v).

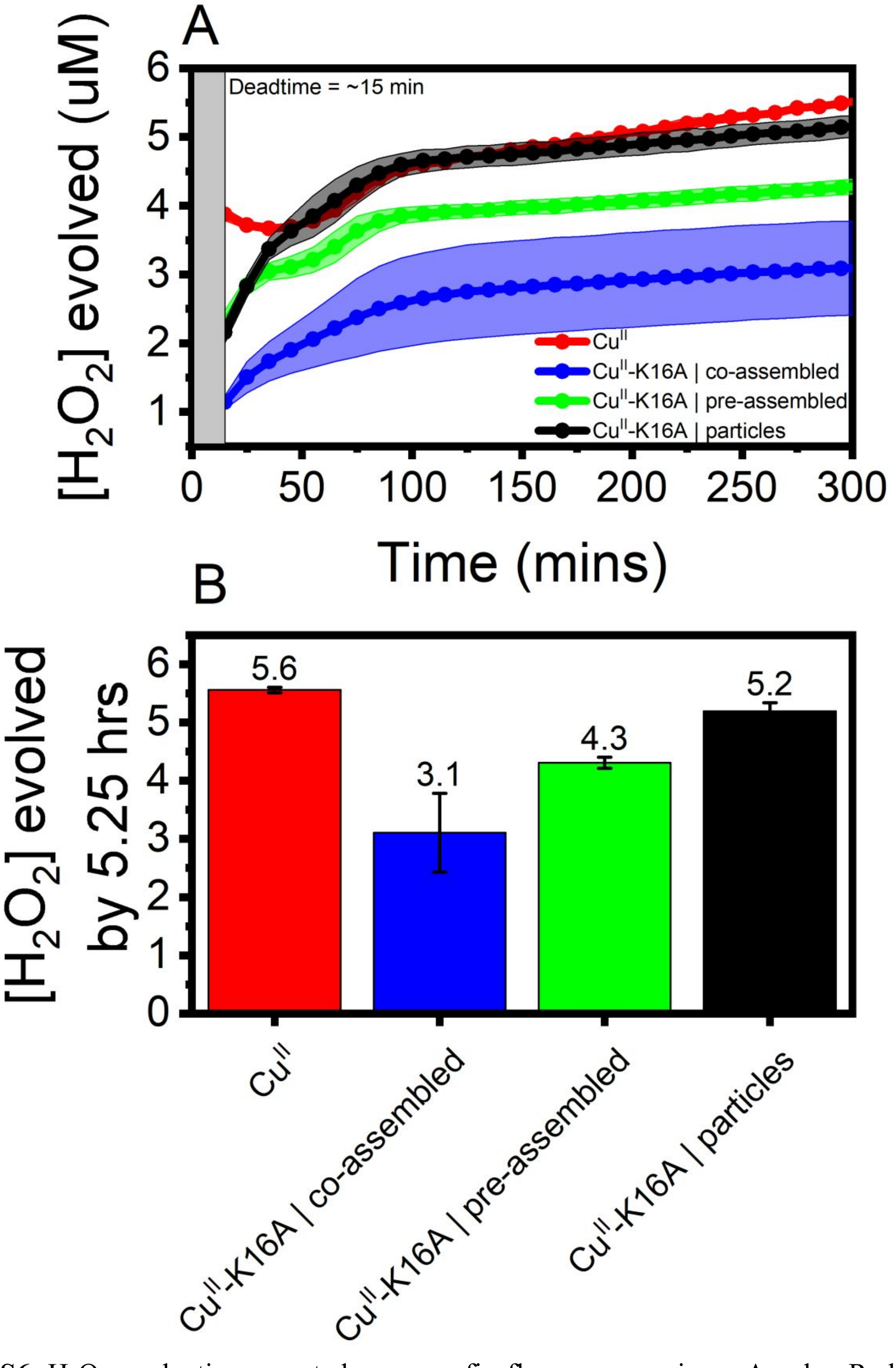

Figure S6. H_2O_2 production reported as resorufin fluorescence via an Amplex Red Assay across co-, pre-, and particulate (unassembled) Cu(II)-K16A preparations (**A**). Cumulative H_2O_2 evolution across measured samples by the final timepoint (**B**). All samples were diluted to a final concentration of 0.1 mM peptide and 0.08 mM Cu(II) via sample preparation.

Scan rate (mV/s)	ΔE_p (mV)	i_{pa}/i_{pc}	D (cm^2/s)	$E_{1/2}$ (mV)
**25**	142	0.79	9.4×10^{-14}	673
**50**	188	0.76	8.3×10^{-14}	671
**100**	194	0.95	1.3×10^{-13}	666
**250**	269	0.94	8.4×10^{-14}	676
**500**	285	0.99	1.0×10^{-13}	663

Table S2. Summary of electrochemical data derived from co-assembled Cu(II)-K16A.

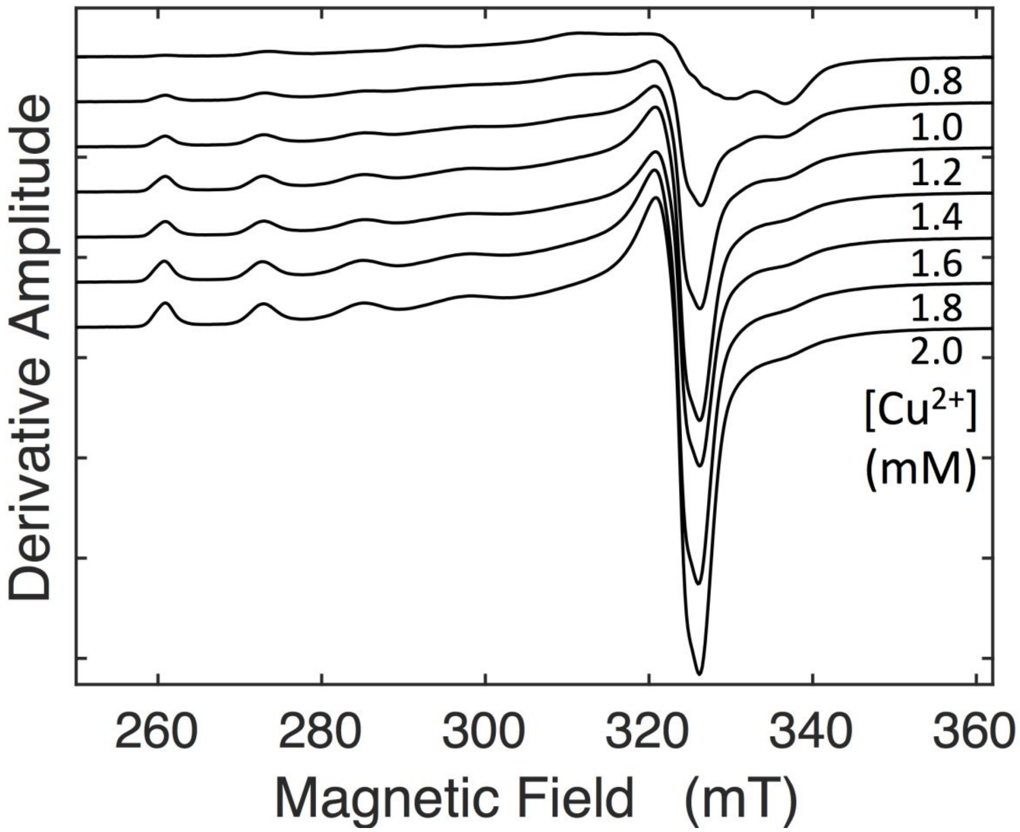

Figure S7. CW-EPR spectra of Cu(II)-K16A for different [Cu(II)]/[K16A] ratios 0.8 ≤ x ≤ 2.0. Each spectrum corresponds to the indicated Cu(II) concentration and 1.0 mM peptide. The absolute (unnormalized) amplitudes of the spectra are referenced to a common scale (arbitrary units, a. u.).

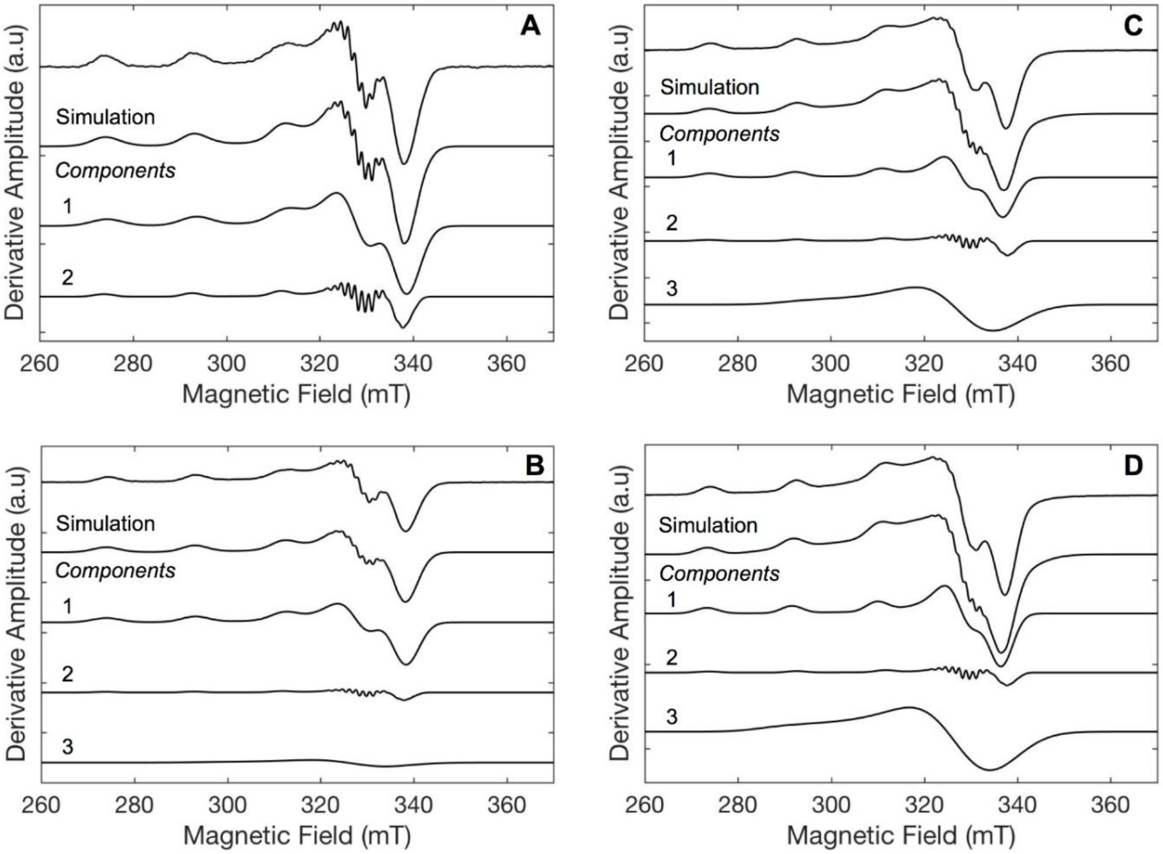

Figure S8. CW-EPR experimental and simulated spectra for the Cu(II)-K16A complex at different [Cu(II)]/[K16A] ratios. The top spectrum in each panel corresponds to the experimental spectrum. The simulated EPR spectrum is the sum of the indicated components, 1-3. The ratio of [Cu(II)]/[K16A] for each panel is: (**A**) 0.1, (**B**) 0.2, (**C**) 0.4, and (**D**) 0.6.

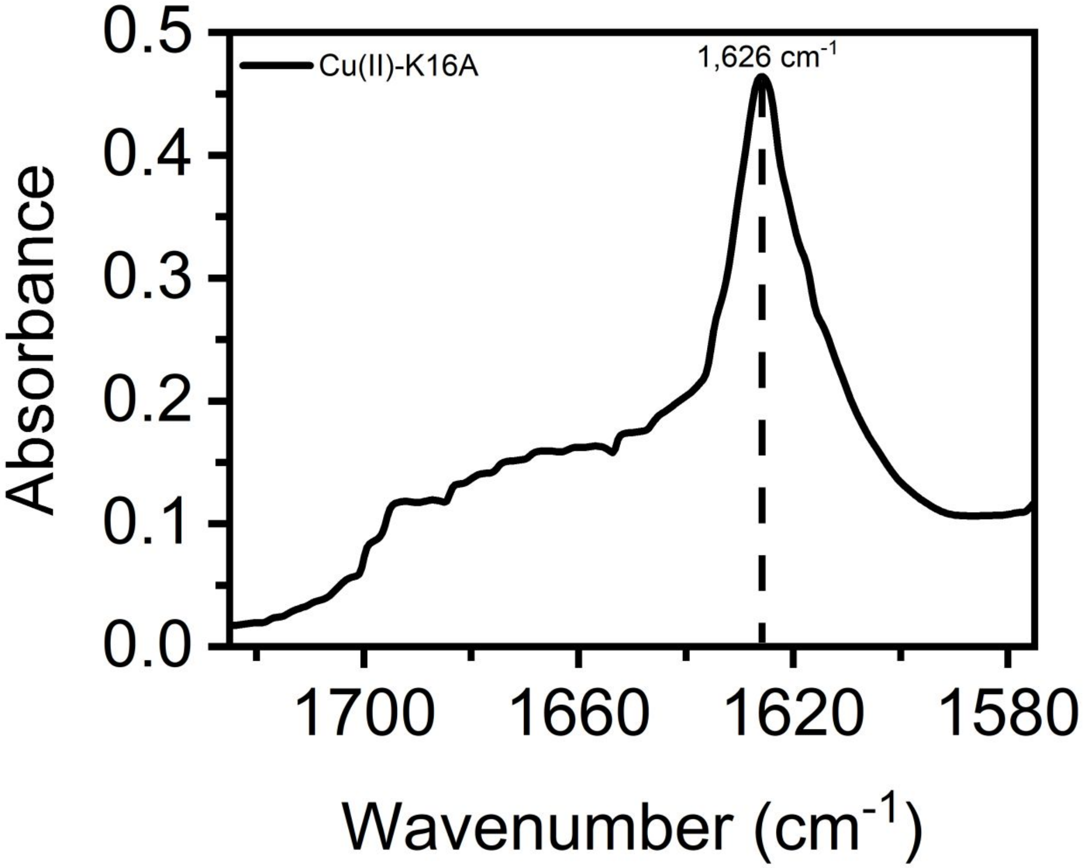

Figure S9. Cu(II)-K16A's vibrational spectrum.

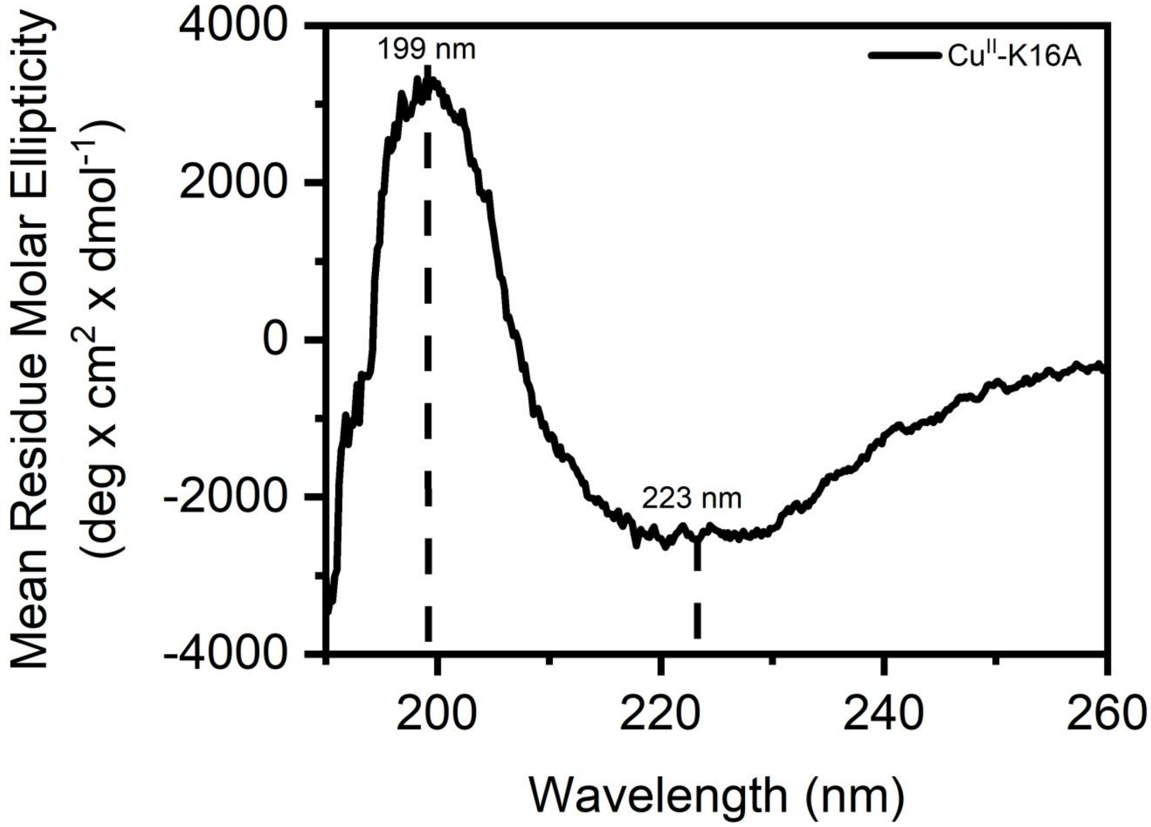

Figure S10. Cu(II)-K16A's circular dichroism spectrum.

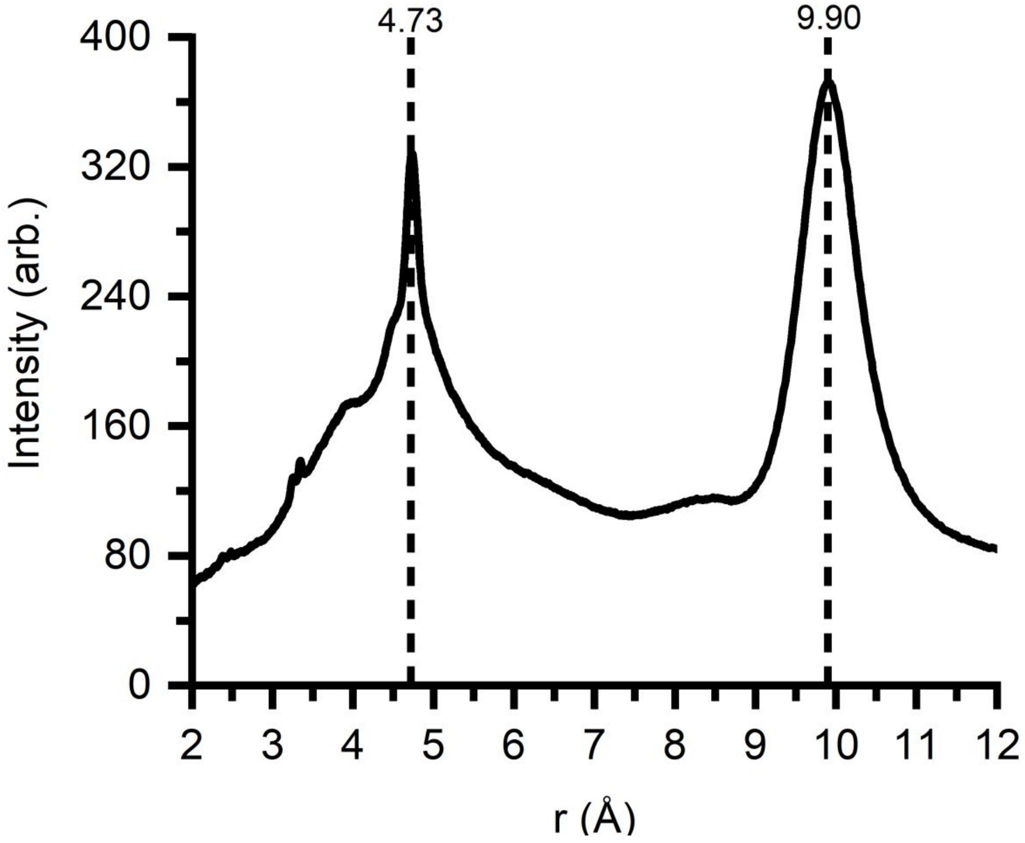

Figure S11. Cu(II)-K16A's pXRD pattern, highlighting amyloid *d*-spacings.

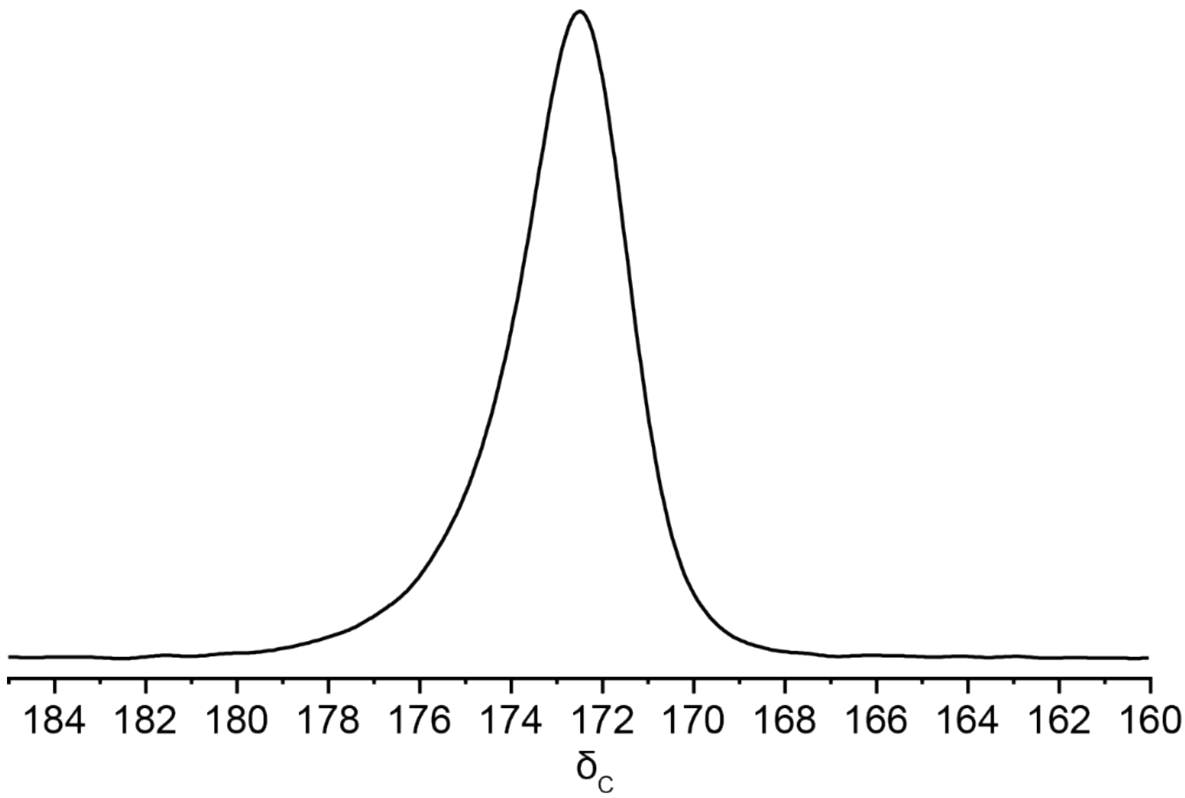

Figure S12. CP-MAS NMR spectra of 1:1 Cu(II)-K16A (HHQ[1-^{13}C]ALV[^{15}N]FFA-NH_2). δ_c = 172.6.

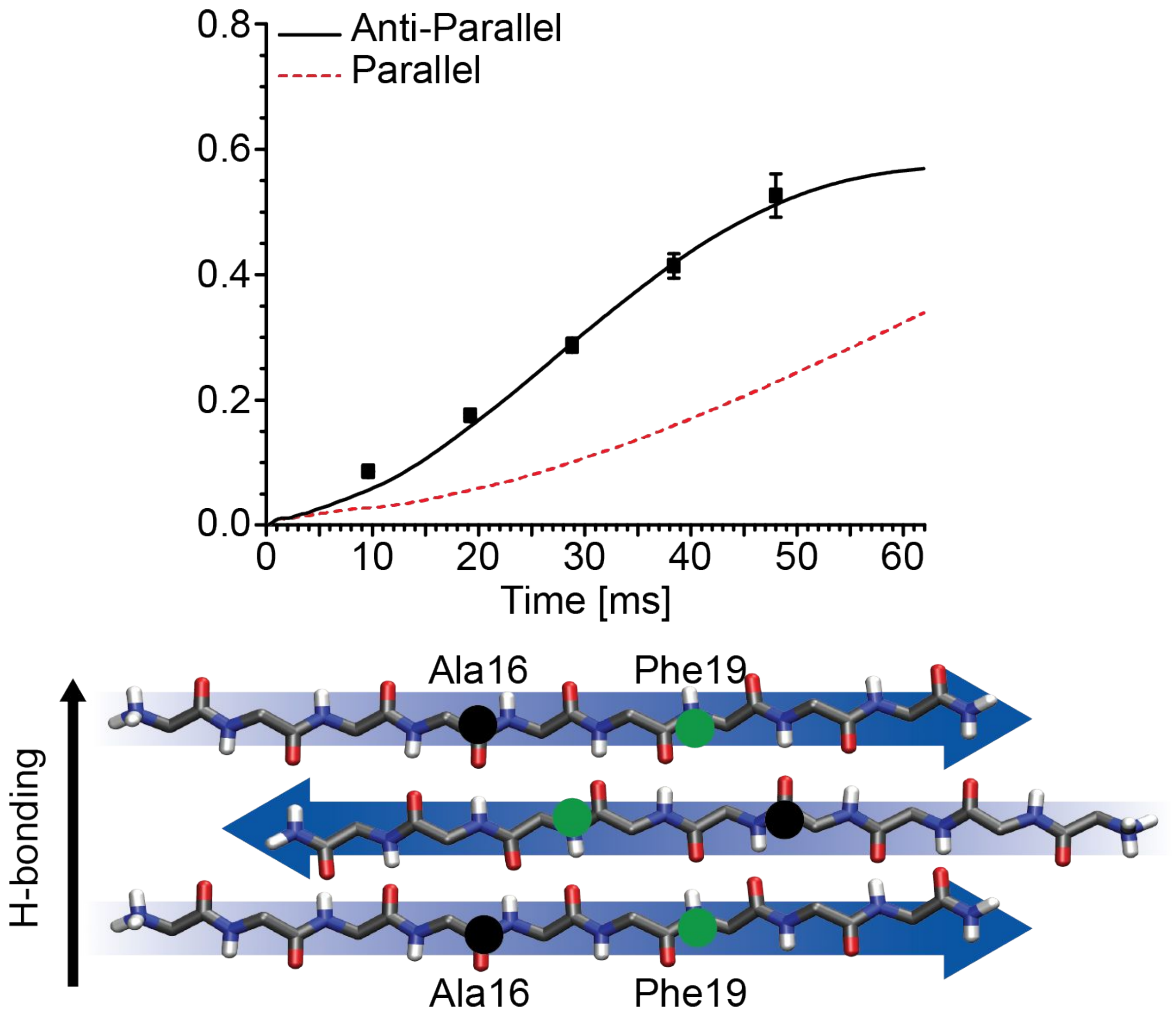

Figure S13. $^{13}C\{^{15}N\}$c REDOR data for HHQ[1-^{13}C]A^{16}LV[^{15}N]F^{19}FA-NH_2 + Cu(II) (Cu(II)-K16A) ribbons fit to a REDOR dephasing curve for antiparallel, doubly out-of-register model (black) as well as an antiparallel in-register model (red dashed). A doubly out-of-register arrangement maximizes access to the histidine sidechains (top). Cartoon of the doubly out-of-register β-sheet with enrichment designations for [1-^{13}C]A^{16} (black) and [^{15}N]F^{19} (green). Sidechains hidden for clarity.

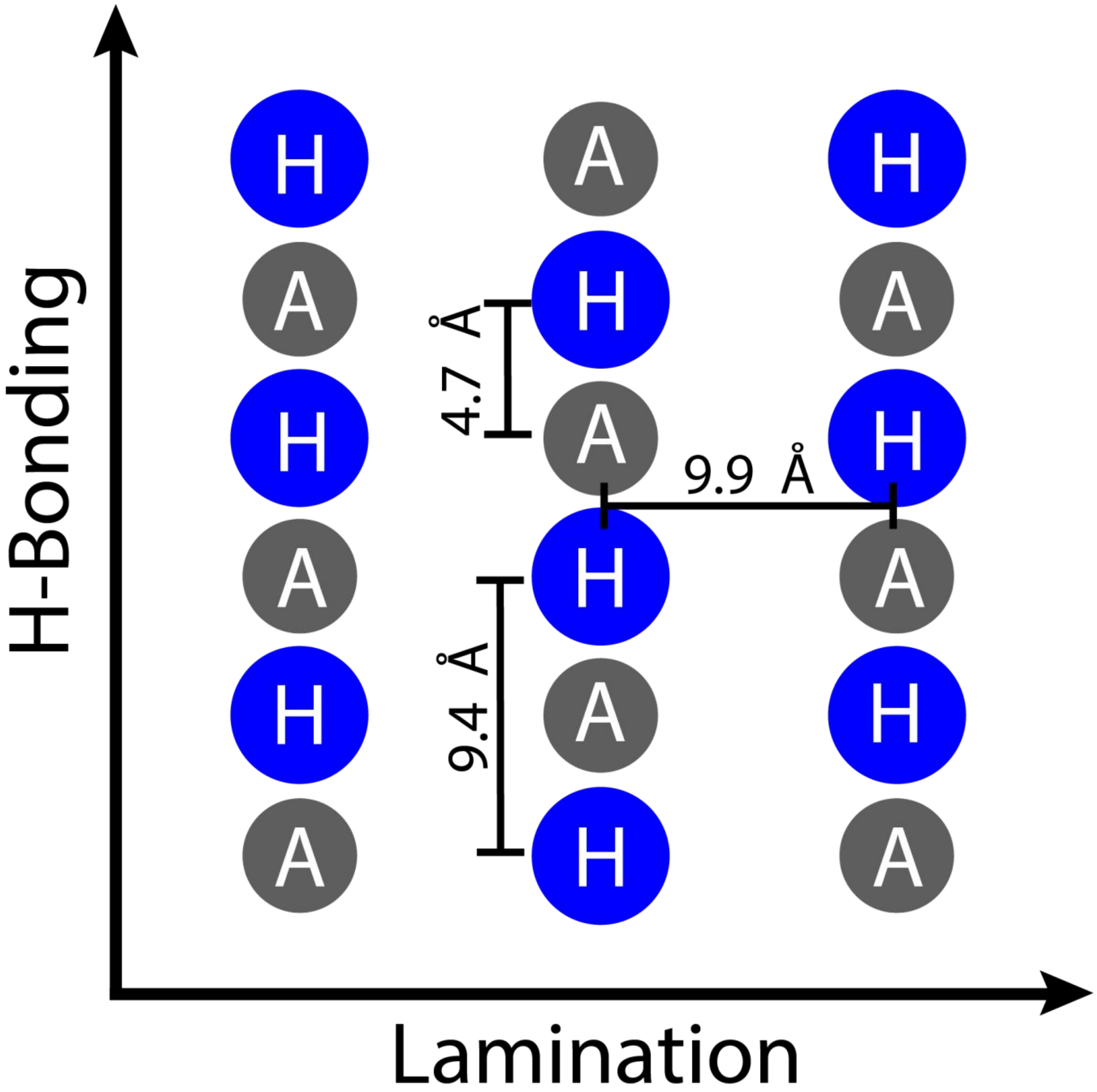

Figure S14. Cartoon highlighting *d*-spacing of N-terminal (H) and C-terminal (A) residues and nearest H-H distance with antiparallel orientation.

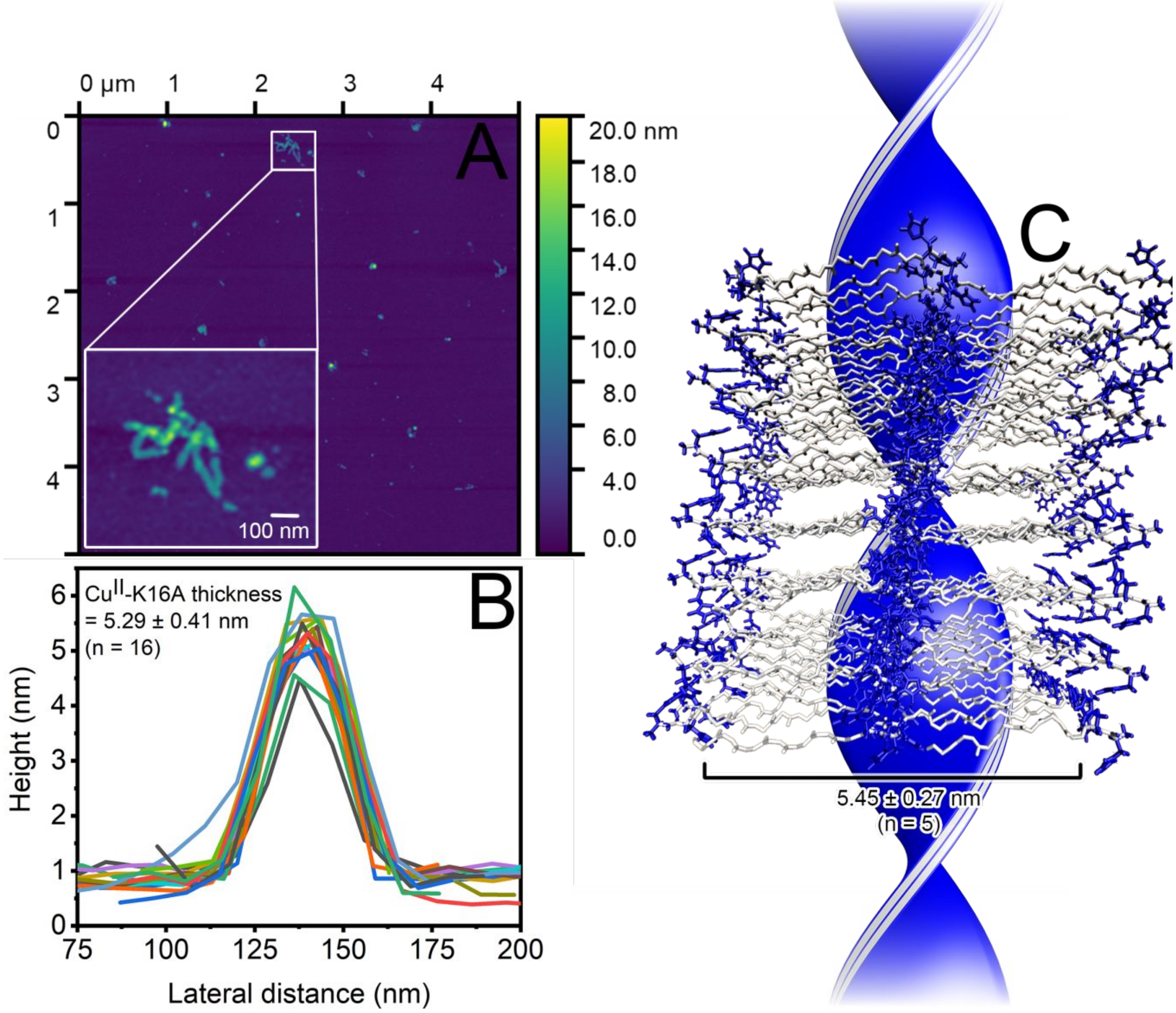

Figure S15. Representative Cu(II)-K16A's atomic force micrograph (**A**) with measurement profiles (**B**). Simulated structural model of apo-K16A's bilayer lattice constructed with experimentally defined *d*-spacings and strand registry (**C**).

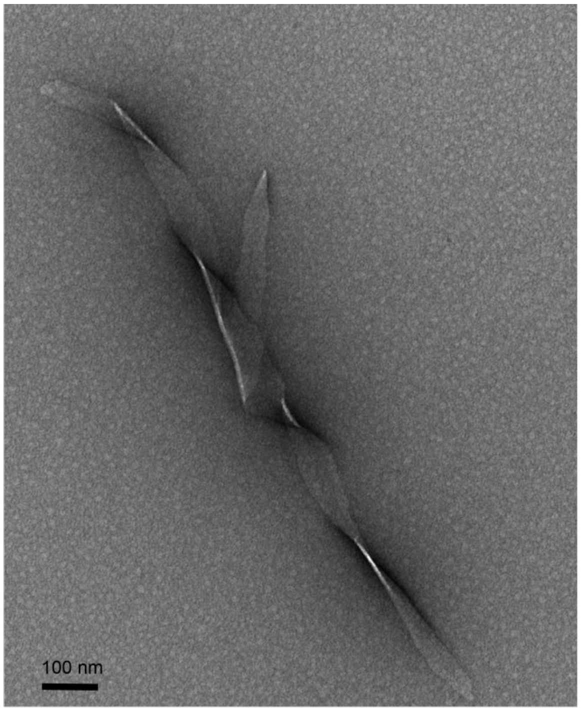

Figure S16. TEM of concentrated (6 mM K16A + 1.2 mM Cu(II)) Cu(II)-K16A prepared for XAFS and ESEEM.

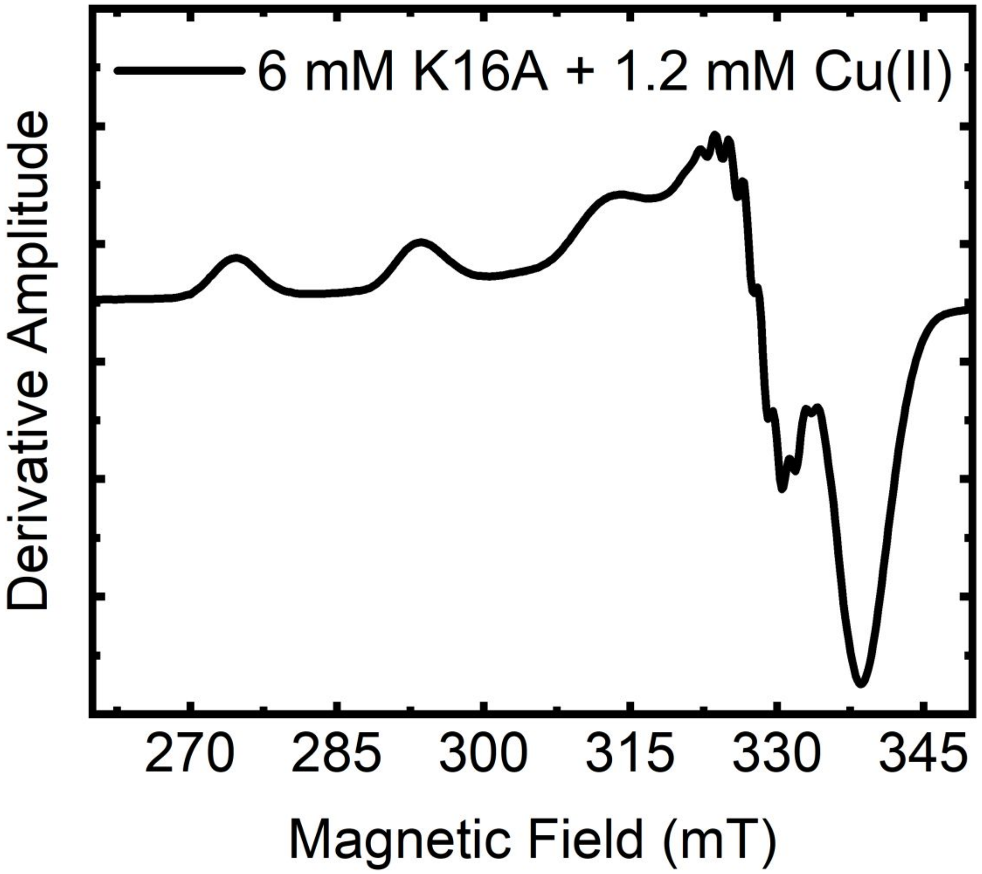

Figure S17. EPR of concentrated Cu(II)-K16A compared with a 1 mM peptide preparation as shown in Fig. 2.

	Fit 1	Fit 2	Fit 3[a]	Fit 4[a]	Fit 5	Fit 6[a]	Fit 7[a]	Fit 8
**Imidazole 1**								
n	3.1(2)	2.0(2)	3	2	1.6(9)	3	2	1.2(9)
r (Å)	1.991(5)	1.968(7)	1.983(6)	2.006(6)	2.051(14)	2.028(7)	2.029(10)	2.028(10)
σ^2 (Å^2)	0.0045(5)	0.0024(4)	0.0055(3)	0.0035(5)	0.004(3)	0.0069(10)	0.005(2)	0.005(3)
**Imidazole 2**								
n	N/A	N/A	N/A	N/A	1.8(10)	1	2	2.0(10)
r (Å)					1.952(4)	1.936(12)	1.944(7)	1.95(1)
σ^2 (Å^2)					0.0021(13)	0.0020(8)	0.0029(7)	0.003(2)
**O-shell**								
n	N/A	1.21(2)	1	2	N/A	N/A	N/A	0.7(3)
r (Å)		2.058(15)	1.98(2)	1.948(9)				2.00(4)
σ^2 (Å^2)		0.005(3)	0.0055(18)	0.009(2)				0.004(2)
**N-shell**								
n	N/A	N/A	N/A	N/A	N/A	N/A	N/A	N/A
r (Å)								
σ^2 (Å^2)								
**ε^2**	1.05	1.03	0.98	0.98	0.92	0.99	0.96	0.86

	Fit 9[a]	Fit 10	Fit 11
**Imidazole 1**			
n	1	N/A	N/A
r (Å)	2.02(1)		
σ^2 (Å^2)	0.004(2)		
**Imidazole 2**			
n	2	N/A	N/A
r (Å)	1.95(1)		
σ^2 (Å^2)	0.004(2)		
**O-shell**			
n	1	N/A	N/A
r (Å)	2.00(2)		
σ^2 (Å^2)	0.004(2)		
**N-shell**			
n	N/A	2.2(2)	4
r (Å)		1.944(4)	1.938(4)
σ^2 (Å^2)		0.0035(12)	0.097(15)
**ε^2**	0.86	1.86	2.22

[a] Number of scatterers restrained to the nearest whole number

Table S3. Statistically valid and chemically reasonable models to the EXAFS data.

Nu #	Type	l	g	also	r	Axx	Ayy	Azz	[redacted]
1	Tensor	1.0000	0.4308	0.0000	2.5000	2.0892	1.8282	0.8660	
2	Tensor	1.0000	0.4308	0.0000	2.5000	2.0892	1.8282	0.8660	
**Nu #**	**Type**	**AEuler1**	**AEuler2**	**AEuler3**	**eeqQ**	**eta**	**QEuler 1**	**QEuler2**	**QEuler 3**
1	Tensor	0.0000	0.0000	0.0000	1.4446	0.9542	81.4721	-102.449	102.983
2	Tensor	-49.4807	-94.4027	-99.1015	1.4446	0.9542	81.4721	-102.449	102.983

Table S4. ESEEM nuclear parameters.

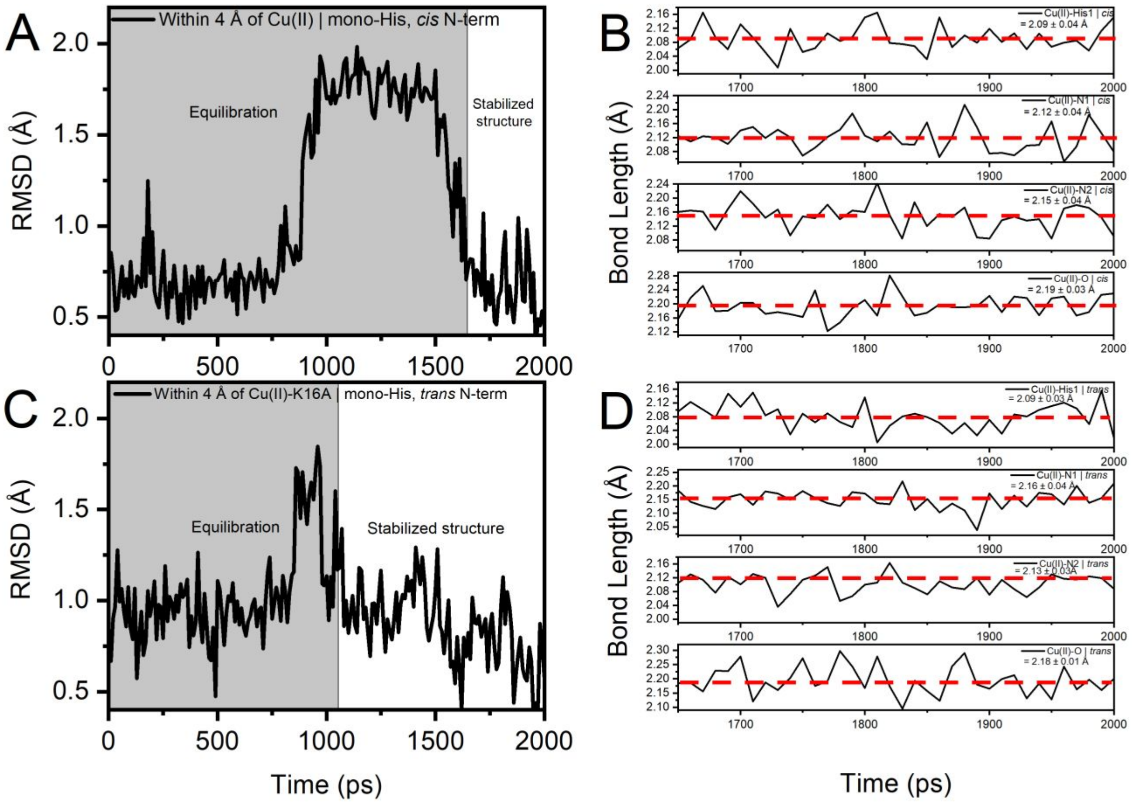

Figure S18. Trajectory overview and dative bond-length statistics for Cu(II)-K16A, mono-His models *cis* (**A-B**) and *trans* (**C-D**). RMSD of simulated the copper center restricted to a 4 Å radius about the DCU, with the frames representing the pre-stabilized and stabilized structure denoted via a gray box. The average bond length values plotted across trajectory measurements in **B** and **D** are represented with a dashed red line. Note: RMSD spike within ~650-1,850 ps for **A** is attributed to a vicinal but non-interacting His ring flip.

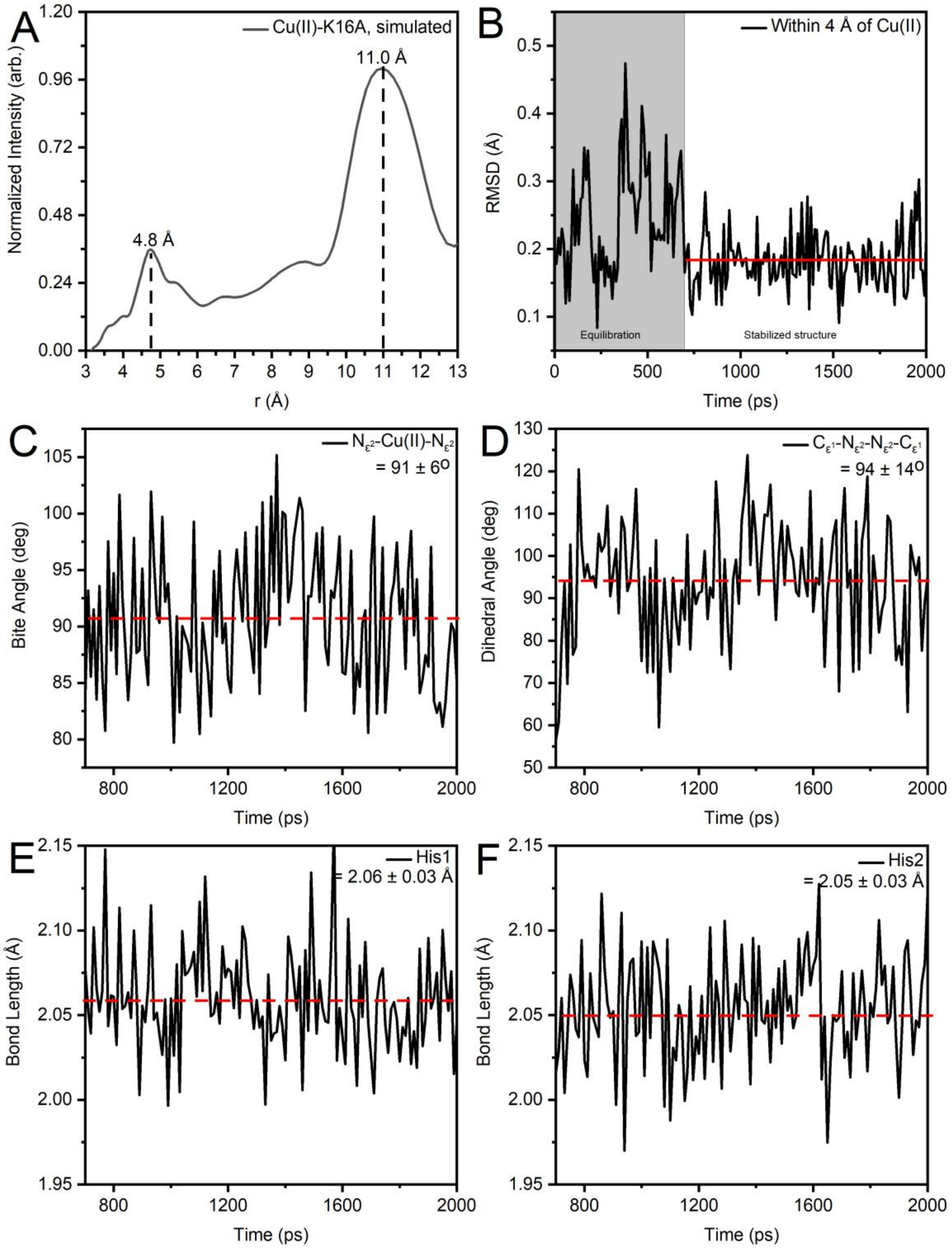

Figure S19. Trajectory overview and dative bond-length statistics for Cu(II)-K16A, bis-*cis*-His. Simulated pXRD pattern of Cu(II)-K16A's coordinates exported from its trajectory's final frame (**A**). RMSD of simulated Cu(II)-K16A lattice, restricted to a 4 Å radius about the DCU, with the frames representing the pre-stabilized and stabilized structure denoted via a gray box (**B**). Ligating histidines' bite (**C**) and dihedral angles (**D**) as a function of time. Both His-Cu(II) bond lengths as a function of time (**E-F**). The average value plotted across trajectory measurements in C-F are represented with a dashed red line.

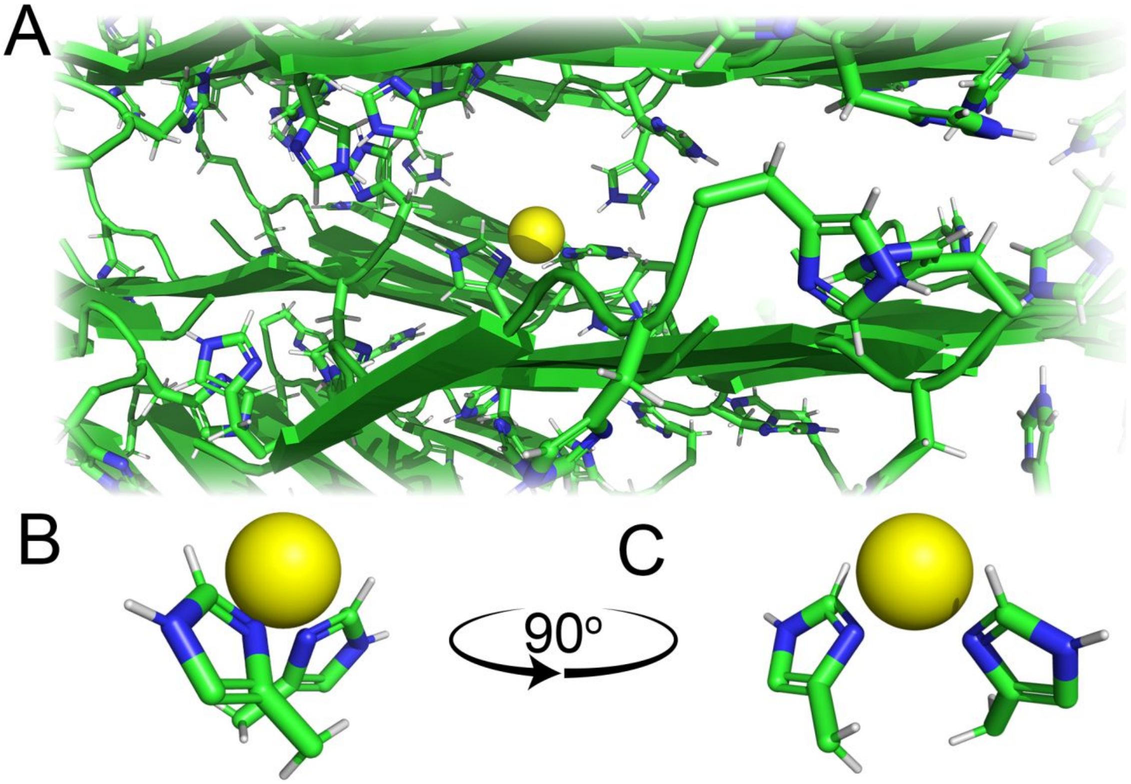

Figure S20. Simulated bis-*cis*-His Cu(II)-K16A assembly with ESEEM and XAFS restraints as a single site featuring a dummy copper atom (**yellow sphere**). Non-histidine sidechains are hidden for clarity (**A**). Time-averaged coordination sphere isolated as a 4 A selection radius consisting of chelating His sidechains only visualized on orthogonal axes (**B-C**).

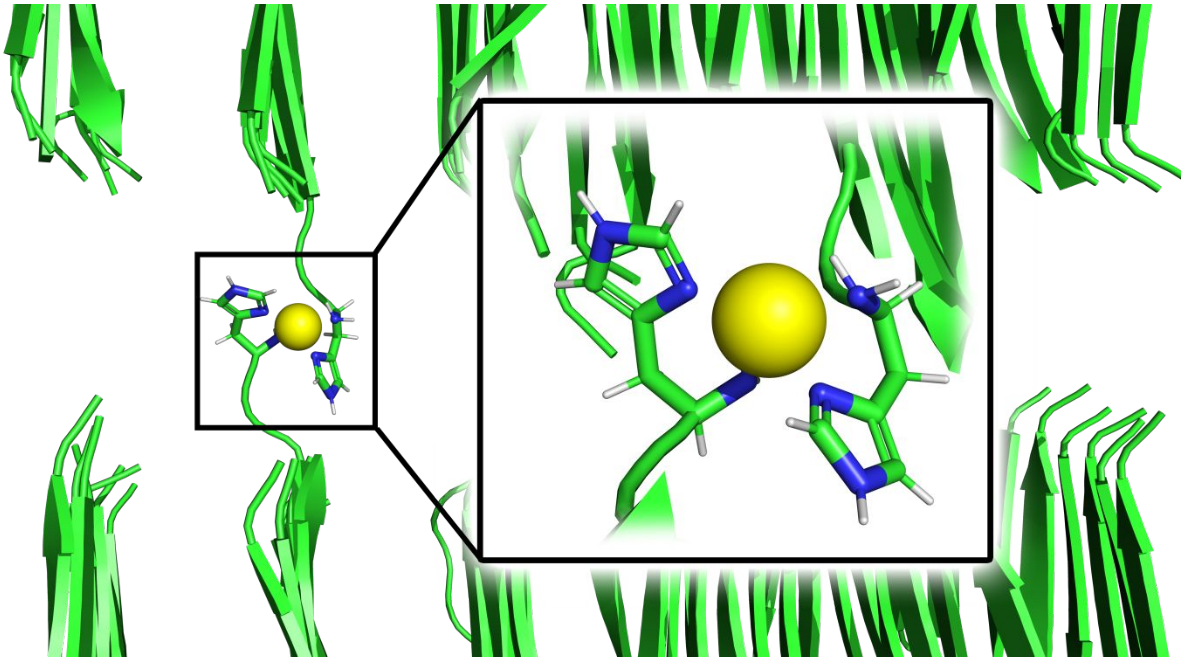

Figure S21. Representative frame and zoom-in (inset) of a simulated DCU with $(Im)_2(ammino)_2$ coordination in a 2 x 8 x 8 peptide lattice with experimental XAFS and ESEEM restraints applied.

Supplementary Video Legends

Supplementary Video 1: Restrained molecular dynamics of Cu(II)-K16A.

Sample trajectory of a 2 x 8 x 8 peptide lattice represented in **Figure 5C** featuring experimental restraints from XAFS and ESEEM on the ligating histidines about a single dummy copper atom (yellow sphere). Non-histidine sidechains are hidden for clarity.

Rate: 10 ps/frame, 0.3 simulated ns/s. Length: 1.3 ns.

Smoothed with thirty passes in PyMOL.

Supplementary Video 2: Two-hundred superimposed frames of a 2 ns Cu(II)-K16A simulation with five dummy Cu(II)s.

The final frame of a 2 ns simulation of a 2 x 8 x 8 peptide lattice rendered as cartoon ribbons, rocked 60^0 about the y axis. The 5 dummy copper atoms (yellow dots) and all histidine ND2 nitrogens (blue dots) are overlaid across all 200 frames, displaying the clustering imposed through the amyloid lattice. Sidechains are hidden for clarity.

REFERENCES

(1) Prenesti, E.; Daniele, P. G.; Prencipe, M.; Ostacoli, G. Spectrum–structure correlation for visible absorption spectra of copper(II) complexes in aqueous solution. *Polyhedron* **1999**, *18* (25), 3233-3241. DOI: https://doi.org/10.1016/S0277-5387(99)00279-X.

(2) Bard, A. J.; Faulkner, L. R. *Electrochemical methods: fundamentals and applications*; John Wiley & Sons, Inc., 2000. Compton, R. G.; Banks, C. E. *Understanding Voltammetry (2nd Edition)*; World Scientific Publishing Company, 2010.

(3) Nečas, D.; Klapetek, P. Gwyddion: an open-source software for SPM data analysis. **2012**, *10* (1), 181. DOI: https://doi.org/10.2478/s11534-011-0096-2.

(4) Gullion, T.; Schaefer, J. Rotational-Echo Double-Resonance NMR. *J. Magn. Reson.* **1989**, *81* (1), 196-200. DOI: 10.1016/0022-2364(89)90280-1. Gullion, T. Introduction to rotational-echo, double-resonance NMR. *Concept Magnetic Res* **1998**, *10* (5), 277-289. DOI: 10.1002/(SICI)1099-0534(1998)10:5<277::AID-CMR1>3.0.CO;2-U.

(5) Li, S.; Mehta, A. K.; Sidorov, A. N.; Orlando, T. M.; Jiang, Z.; Anthony, N. R.; Lynn, D. G. Design of Asymmetric Peptide Bilayer Membranes. *J. Am. Chem. Soc.* **2016**, *138* (10), 3579-3586. DOI: 10.1021/jacs.6b00977.

(6) Kraft, S.; Stümpel, J.; Becker, P.; Kuetgens, U. High resolution x-ray absorption spectroscopy with absolute energy calibration for the determination of absorption edge energies. *Rev. Sci. Instrum.* **1996**, *67* (3), 681-687. DOI: 10.1063/1.1146657.

(7) ATHENA, ARTEMIS, HEPHAESTUS: data analysis for X-ray absorption spectroscopy using IFEFFIT. *J. Synchrotron Radiat.* **2005**, *12* (4), 537-541. DOI: 10.1107/S0909049505012719.

(8) *Scarrow, R. S.; Shearer, J. EXAFS123, v. 0.5; Trinity University: San Antonio, TX, 2019.*; (accessed. Rehr, J. J.; Kas, J. J.; Vila, F. D.; Prange, M. P.; Jorissen, K. Parameter-free calculations of X-ray spectra with FEFF9. *Phys. Chem. Chem. Phys.* **2010**, *12* (21), 5503-5513, 10.1039/B926434E. DOI: 10.1039/B926434E.

(9) Shearer, J.; Soh, P. The Copper(II) Adduct of the Unstructured Region of the Amyloidogenic Fragment Derived from the Human Prion Protein is Redox-Active at Physiological pH. *Inorg. Chem.* **2007**, *46* (3), 710-719. DOI: 10.1021/ic061236s.

(10) **Schrödinger Release 2020-1:** Maestro, Schrödinger, LLC, New York, NY, 2020.

(11) Berendsen, H. J. C.; van der Spoel, D.; van Drunen, R. GROMACS: A message-passing parallel molecular dynamics implementation. *Comput. Phys. Commun.* **1995**, *91* (1), 43-56. DOI: https://doi.org/10.1016/0010-4655(95)00042-E.

(12) Jorgensen, W. L.; Chandrasekhar, J.; Madura, J. D.; Impey, R. W.; Klein, M. L. Comparison of simple potential functions for simulating liquid water. *J. Chem. Phys.* **1983**, *79* (2), 926-935. DOI: 10.1063/1.445869 (acccessed 2020/05/28).

(13) Jorgensen, W. L.; Maxwell, D. S.; Tirado-Rives, J. Development and Testing of the OPLS All-Atom Force Field on Conformational Energetics and Properties of Organic Liquids. *J. Am. Chem. Soc.* **1996**, *118* (45), 11225-11236. DOI: 10.1021/ja9621760.

(14) Verlet, L. Computer" experiments" on classical fluids. I. Thermodynamical properties of Lennard-Jones molecules. *Phys. Rev.* **1967**, *159* (1), 98.

(15) Berendsen, H. J. C.; Postma, J. P. M.; Gunsteren, W. F. v.; DiNola, A.; Haak, J. R. Molecular dynamics with coupling to an external bath. *J. Chem. Phys.* **1984**, *81* (8), 3684-3690. DOI: 10.1063/1.448118.

(16) Parrinello, M.; Rahman, A. Polymorphic transitions in single crystals: A new molecular dynamics method. *J. Appl. Phys.* **1981**, *52* (12), 7182-7190. DOI: 10.1063/1.328693.

(17) Hess, B.; Bekker, H.; Berendsen, H. J. C.; Fraaije, J. G. E. M. LINCS: A linear constraint solver for molecular simulations. *J. Comput. Chem.* **1997**, *18* (12), 1463-1472. DOI: 10.1002/(sici)1096-987x(199709)18:12<1463::Aid-jcc4>3.0.Co;2-h. Hess, B. P-LINCS: A Parallel Linear Constraint Solver for Molecular Simulation. *J. Chem. Theory Comput.* **2008**, *4* (1), 116-122. DOI: 10.1021/ct700200b.

(18) Braun, S.; Humphreys, C.; Fraser, E.; Brancale, A.; Bochtler, M.; Dale, T. C. Amyloid-associated nucleic acid hybridisation. *PloS one* **2011**, *6* (5), e19125, Research Support, Non-U.S. Gov't. DOI: 10.1371/journal.pone.0019125.
(19) **The PyMOL Molecular Graphics Syste, Version 2.0**, Schrodinger, LLC.
(20) Svergun, D.; Barberato, C.; Koch, M. H. J. CRYSOL - A program to evaluate x-ray solution scattering of biological macromolecules from atomic coordinates. *J. Appl. Crystallogr.* **1995**, *28*, 768-773. Franke, D.; Petoukhov, M. V.; Konarev, P. V.; Panjkovich, A.; Tuukkanen, A.; Mertens, H. D. T.; Kikhney, A. G.; Hajizadeh, N. R.; Franklin, J. M.; Jeffries, C. M.; et al. ATSAS 2.8: a comprehensive data analysis suite for small-angle scattering from macromolecular solutions. *J. Appl. Crystallogr.* **2017**, *50* (4), 1212-1225. DOI: doi:10.1107/S1600576717007786.
(21) Sun, L.; Savory, J. J.; Warncke, K. Design and implementation of an FPGA-based timing pulse programmer for pulsed-electron paramagnetic resonance applications. *Concepts Magn. Reson. B: Magn. Reson. Eng.* **2013**, *43* (3), 100-109.
(22) Fauth, J.-M.; Schweiger, A.; Braunschweiler, L.; Forrer, J.; Ernst, R. R. Elmination of unwanted echoes and reduction of dead time in three-pulse electron spin-echo spectroscopy. *J. Magn. Reson.* **1986**, *66*, 74-85.
(23) Peisach, J.; Mims, W. B.; Davis, J. L. Studies of the electron-nuclear coupling between Fe(III) and N-14 in cytochrome-P-450 and in a series of low-spin heme compounds. *J. Biol. Chem.* **1979**, *254* (24), 2379-2389.
(24) Mims, W. B. Elimination of the dead-time artifact in electron spin-echo envelope spectra. *J. Magn. Reson.* **1984**, *59*, 291-306.
(25) Sun, L.; Hernandez-Guzman, J.; Warncke, K. OPTESIM, a versatile toolbox for numerical simulation of electron spin echo envelope modulation (ESEEM) that features hybrid optimization and statistical assessment of parameters. *J. Magn. Reson.* **2009**, *200*, 21-28 (Cover Article). DOI: 10.1016/j.jmr.2009.05.012.
(26) Hernández-Guzmán, J.; Sun, L.; Mehta, A. K.; Dong, J.; Lynn, D. G.; Warncke, K. Copper(II)-bis-Histidine Coordination Structure in a Fibrillar Amyloid β-Peptide Fragment and Model Complexes Revealed by Electron Spin Echo Envelope Modulation Spectroscopy. *ChemBioChem* **2013**, *14* (14), 1762-1771. DOI: 10.1002/cbic.201300236.
